# Design and maintenance of community-based cohorts in sub-Saharan Africa: a longitudinal evaluation of participant attrition in SchistoTrack

**DOI:** 10.1136/bmjph-2024-002151

**Published:** 2025-08-10

**Authors:** Christin Puthur, Betty Nabatte, Benjamin Tinkitina, Narcis B Kabatereine, Goylette F Chami

**Affiliations:** 1 Big Data Institute, Nuffield Department of Population Health, University of Oxford, Oxford, UK; 2 Division of Vector Borne and Neglected Tropical Diseases, Republic of Uganda Ministry of Health, Kampala, Uganda

**Keywords:** Epidemiologic Methods, Statistics as Topic, Community Health, Communicable Disease Control, Public Health

## Abstract

**Introduction:**

Understanding participant attrition in longitudinal studies is essential for maintaining cohorts, establishing targeted interventions and assessing potential biases introduced in study analyses. Yet, limited metrics, models and long-term assessments exist for the design and evaluation of community-based cohorts in sub-Saharan Africa.

**Methods:**

We prospectively assessed participant attrition in the SchistoTrack cohort. A total of 2885 individuals aged 5–92 years, who were recruited in 2022, were examined from 1445 randomly sampled households across three rural Ugandan districts. Baseline data on sociodemographics, medical history, spatial factors and clinical examinations were collected, with annual and seasonal follow-ups to 2025. Profiles of attriters and rejoiners were established with logistic regressions. Community engagement, retention strategies and study expansion to 4321 participants were evaluated.

**Results:**

Overall attrition rates were stable across the years, ranging from 20.9% to 27.7%. Attriter profiles were established within the first year and were stable predictors of the recurrent attrition. Older individuals were less likely (OR 0.987, 95% CI 0.983 to 0.991), whereas more educated individuals were more likely to be lost to follow-up (OR 1.086, 95% CI 1.057 to 1.117). Individuals belonging to larger households and homes closer to government health centres were less likely to have an attrition event (ORs 0.920–0.924). Fishermen were not more likely than other individuals to have an attrition event, either overall or seasonally. 63.3% (374/591) of participants who dropped out from the first major follow-up and 54.1% (725/1341) of all participants who had any loss to follow-up for reasons other than death later rejoined the study. Schistosome infection and medical referrals were not associated with later attrition. Communicating clinical findings, adjusting incentives and study logistics did not influence attrition.

**Conclusions:**

By providing metrics and models for tracking attrition, our attrition analysis framework can guide the design and evaluation of community-based cohorts in rural sub-Saharan Africa.

**WHAT IS ALREADY KNOWN ON THIS TOPIC**

- Participant attrition in longitudinal studies is common and, if not measured and accounted for, can lead to analytical biases and reduced statistical power to produce substandard study designs as well as reduced access to continued care for participants needing further treatment.

**WHAT THIS STUDY ADDS**

- We comprehensively tracked attrition in a large-scale prospective cohort (SchistoTrack). Attrition at the levels of individuals, households, villages and districts was examined in rural Uganda within highly mobile fishing communities. We show how periodic recruitment (study expansions), effective community engagement, expectations of participants rejoining after loss to follow-up (as opposed to being true dropouts) and adequate participant incentives, among other study design considerations, help to manage attrition and its impact on cohort studies.

**HOW THIS STUDY MIGHT AFFECT RESEARCH, PRACTICE OR POLICY**

- We challenge assumptions about hard-to-reach populations, encouraging cohort development for understudied groups or contexts. The attrition observed here can also be used more widely to design effective participant sampling and sample size calculations across different longitudinal study designs. If replicated elsewhere in sub-Saharan Africa, the framework of SchistoTrack may help guide the rigorous design and maintenance of community-based cohorts in this region.

## Background

 Longitudinal studies (cohorts) in epidemiology are essential for the identification of key risk factors of disease without reverse causality and to understand disease progression. Participant recruitment and retention underpin the success of any longitudinal study trying to ascertain individual or population-level trends. Three main ways in which attrition could statistically affect results from a longitudinal study include reduced statistical power, reduced generalisability and selection bias.^1^ High attrition can lead to reduced analytical power and the ability to detect small effects. Non-random attrition reduces the generalisability of study findings by potentially compromising the initial representativeness of the study population. Participant characteristics associated with both attrition and study outcomes may lead to selection biases in the estimates of outcomes.^2 3^ Importantly, high rates of attrition may be an indicator of ineffective community engagement or diminished trust and receptiveness of study participants.^4^ At worst, attrition could impact participant care needs, including treatment and medical referrals directly provided by a study or as a result of study coordination with the local health system.^5^ Yet, attrition in community-based cohort studies in sub-Saharan Africa (SSA), especially studies focused on prospective analyses of individual-based outcomes, remains poorly studied despite the urgent need for more cohort studies in these areas.^6 7^

In SSA, the study of attrition has been focused on non-adherence or loss to follow-up (LTFU) in specialist populations to monitor the treatment of patients with tuberculosis (TB)^8^ or antiretroviral treatment (ART) administration for people living with HIV (PLHIV). A systematic review and meta-analysis of risk factors associated with LTFU and non-adherence to ART in low- and middle-income countries,^9^ which included 87.4% of studies from SSA, found that the percentage of patients identified as LTFU varied widely, ranging from 2.8% to 65.6%. This wide range poses challenges for sample size estimations when designing cohorts. Most studies in the review had a follow-up period of only 6–24 months, with LTFU defined as being away from care for a minimum period ranging from 30 to 90 days. Studies with multiple follow-up timepoints only reported annual attrition and did not consider participants who might have rejoined after missing a follow-up. It is unclear how attrition observed from health facilities generalises to community-based studies.

A wide range of participant characteristics that vary across studies have been associated with an increased likelihood of LTFU for ART adherence.^9^ These characteristics included male sex, younger age, lower educational attainment, being single, unemployment, advanced clinical stage, low weight, poor functional status, poor ART adherence and participant non-disclosure of HIV-positive status. The LTFU characteristics have been used during ART initiation to screen participants likely to be LTFU for targeted interventions. It remains an open question as to whether such characteristics are generalisable beyond specialist populations of PLHIV. In contrast, in fields with possibly fewer financial resources for follow-up, such as malaria, migration was a major reason for attrition.^10^ Apart from studies on TB and HIV, participants lost at the first follow-up often are excluded from later study timepoints.^11^ Community-based studies in SSA often lack an assessment of factors statistically associated with attrition unless the goal of the study is to assess a population for potential vaccine trials.^12^ There is a need to gather information on how study design and participant characteristics may influence attrition to address how best to stratify initial recruitment or to determine which participants may later require the development of targeted retention strategies.

We conducted a comprehensive analysis of attrition in the prospective community-based cohort, SchistoTrack. This study has annual and seasonal follow-ups and explores coinfection interactions and concurrent long-term health conditions associated with schistosomiasis. Attrition was assessed over 4 years for 2885 individuals aged 5–92 years from 38 villages in Western and Eastern Uganda. The aims of this study were to provide attrition metrics and describe patterns of attrition across diverse populations in rural Uganda in order to provide a framework for the design and evaluation of community-based cohorts.

## Materials and methods

### Study context

This study was nested within SchistoTrack.^13^ This ongoing cohort was established in 2022 in three rural districts of Pakwach, Buliisa and Mayuge along the River Nile, Lake Albert and Lake Victoria in Uganda, respectively. At the time of the study, Pakwach was the only study district with no hospitals and only primary healthcare centres.^14^ Informal private drug shops were commonly available within or near all study villages. The study districts have received mass drug administration with praziquantel since 2003.^15^

### Participant sampling and prospective study design

**Figure 1:**
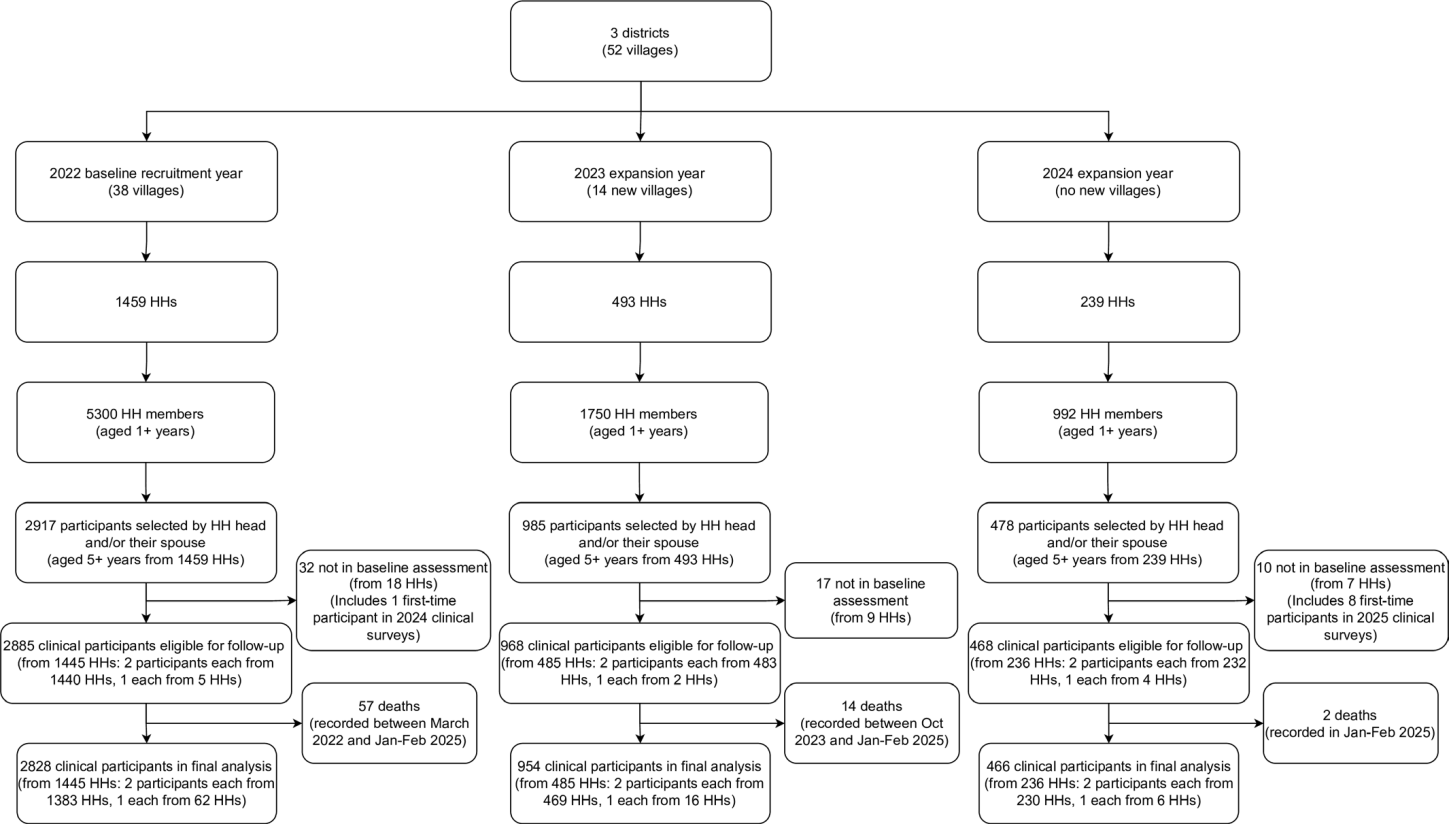
Participant selection flow diagram. Our analysis considered the 4321 individuals, both recruited and clinically assessed at baseline, to constitute the total cohort participants. However, these numbers would rise to 4330 if recruited individuals who were clinically assessed at a later timepoint were included. The total number of clinical participants in the study, including deaths, was 2885 in the year 2022, 3853 in 2023, 4322 in 2024 and 4330 in 2025. HHs, households.

Across the entire cohort, 4380 individuals from 2191 households (HHs) were recruited (figure 1). In January 2022, 1459 HHs were randomly selected with oversampling from 38 rural villages. Random numbers from one to the total number of HHs in the village were generated with uniform probability to sample 40 HHs and 80 individuals per village. For villages that were small (*<*100 HHs), two adjacent villages were combined to form one village sampling unit. At baseline, four villages were combined, leaving a total of 36 village sampling units. Local registers were used that were maintained by the local council (village government) and ordered by year of HH settlement. Oversampling of 30 HHs was done, and these additional HHs were selected if the originally sampled HH was no longer in the village.^15^ HHs were required to have been settled in the villages for at least 6 months out of the year. Within each HH, the head and/or their spouse selected one adult (18+ years) and one child (5+ years) to participate in a clinical study.

A total of 2885 individuals were recruited at baseline in 2022. Treatment with praziquantel was provided at each annual study visit, irrespective of schistosomiasis diagnosis. A short-term drug efficacy follow-up was conducted 3–4 weeks after baseline (March 2022) to evaluate the efficacy of the praziquantel treatment on infection status and intensity. Annual follow-up examinations were carried out in January–February 2023, January–February 2024 and January–February 2025. These three timepoints represent the dry seasons in the study districts, with the January–February 2024 timepoint experiencing longer rains from the preceding rainy season.^16^ Additionally, a follow-up examination was conducted in October 2023 to assess the seasonal variation (here the rainy season) in schistosome and malaria infections.^17^ To enable analyses of population trends for smaller effect sizes, two cohort expansions were conducted in 2023 with 968 participants recruited from 14 new villages (12 new village sampling units) and in 2024 with 468 participants recruited from previously included study villages (figure 1). Expansions were done at the time of follow-up of already recruited participants. The aim of the expansions was to achieve a target number of no less than 65 individuals per village sampling unit to reach a minimum of 3120 individuals seen each year and expand the participant sampling frame to reach those targets, considering attrition and rejoining. The target was set to incorporate anticipated smaller effect sizes of new infection exposures (eg, hepatitis B and HIV) as well as to enable the study of coinfection interactions at the population level.^15^ Past participants were not replaced, as requested by study communities. Expansion strategies were decided together with district and village leaders in response to non-participant requests to join the study after the baseline timepoint.

### Community-based data collection

Digital surveys were developed using Open Data Kit (ODK) Collect V.2022.4.2 and ODK Collect V.2023.2.4. Data entry through ODK surveys was done using Android tablets with Android V.9.0 Pie. Sociodemographic information of all members aged 1 year and above of the sampled HHs was collected by surveyors at the time of recruitment. Surveyors provided containers for stool and urine samples to village health team members (VHTs) for distribution to the HH participants selected for the clinical study. VHTs and local village leaders mobilised study participants. SchistoTrack teams spent 2 days in each village to conduct clinical examinations at a central location, such as a school or church that could serve/border two villages.

### Community engagement and health outreach

Community engagement was conducted before every timepoint. District sensitisation and mobilisation of participants were led by district focal people, community leaders and VHTs through community meetings. During the baseline examinations in 2022, all adult participants received 10 000 Ugandan Shillings (approximately US$3) as compensation, while children were given pens and notebooks. At the follow-up timepoints, large 1 kg soap bars were provided in place of cash as incentives to each HH. Participants were informed about the type of compensation during mobilisation before the study timepoint. Participants were also informed of their schistosome infection status from their last visit.

Technicians, nurses and sonographers examined participants. Clinical stations included separate areas for stool and urine sample drop-off, blood tests, nurse anthropometry, nurse palpation, ultrasonography, and food, drink, praziquantel and antimalarial treatment. The treatment was provided by local government nurses as part of the study outreach activities. Participants in need of further medical attention were provided medical referrals, on-site consultations with local health workers from their districts (nurses and doctors to facilitate referrals) and transported to local facilities for serious cases. The study also coordinated with any other local doctors available within health facilities, followed up on the care of participants and, where needed, financially supported direct costs of additional diagnostic tests that were not available in government health facilities. Direct feedback on the study design and issues related to attrition was elicited not only at each timepoint but also in community engagement forums conducted in December 2023 with all adult participants within their villages.

### Outcomes

Our attrition outcomes focus on four major study timepoints, including the first annual follow-up of January–February 2023, the seasonal timepoint of October 2023, the second annual follow-up of January–February 2024 and the third annual follow-up of January–February 2025. Reasons for attrition were communicated to the study team by VHTs and informed by knowledge of VHTs and village leaders, as well as directly from study participants or their HH members if available during the timepoint.

To identify factors associated with attrition, participants were classified as non-attrited if they attended all timepoints under consideration and as attrited if they missed one or more study timepoints. Secondary outcomes included total attrition across all major study timepoints; true dropout, that is, participation at only the baseline study timepoint; rejoining, that is, study participation after an attrition event; and attrition count, that is, the number of attrition events for a participant. Recurrent attrition, defined as a longitudinal recurrent event with a binary variable indicating at each major study follow-up whether there was loss to follow-up for every individual, was also examined. The attrition status was ultimately decided by observation from the study teams, noting whether participants were seen at any clinical stations.

### Covariates

The history of disease and sociodemographic variables were collected using an HH survey administered by local surveyors fluent in Lusoga, Lugungu or Alur and English at the time of baseline recruitment. Spatial variables were defined using waypoint data collected with the HH survey, as well as waypoints collected from all private drug shops and health centres located within the study catchment in 2022.

Detailed definitions of all variables are provided in the online supplemental file 1. Sociodemographic variables of age, sex, tribe, majority religion, education, occupation and primary school enrolment of children (*<*18 years) were measured at the individual level. At the HH level, variables measured included home quality, social status, HH size (total members), number of deaths in the past 3 years, years of settlement in the village, home ownership, number of rooms in the home, drinking water source, drinking water treatment/safety and the level of improved sanitation and hygiene facilities. Social status was defined as any adult HH member holding or previously having a position in the local council, village health team, religious or clan leadership or influential beach management committees, and conferred to all individuals within the HH. To examine the access to care for the participant outside of the cohort study, spatial factors included HH distance (in km) to the nearest drug shop and government health centre. For the district, Mayuge was set as the reference to look at geographical differences between Western (Pakwach and Buliisa) and Eastern (Mayuge) Uganda, where there are many more high-level or specialist health facilities.^14^

### Statistical analysis

All analyses were compiled in R V.4.2.1. Participant characteristics and attrition metrics are provided for all individuals recruited into the cohort. For understanding any potential sources of non-random attrition, models of attrition were built for the 2828 individuals recruited at baseline (with deaths excluded) to provide a longitudinal analysis of attrition. Logistic regressions were performed at the individual level. For the main outcome of overall attrition, the best-fit parsimonious model was selected using backward elimination based on the Bayesian Information Criterion.^18^ Keeping the variable selection the same after the main outcome model to enable cross-model comparisons, logistic regressions were also run for each major timepoint, for true dropouts and for rejoiners. Given the large number of individuals with no attrition events, the total number of attrition events was studied in a negative binomial regression. To account for the paired sampling design within HHs, SEs were clustered in the logistic and negative binomial regressions at the HH level.^19^ For recurrent attrition, intraclass correlation coefficients (ICCs) for empty multilevel models (MLMs) with individual, HH and village levels were investigated to assess within-cluster and between-cluster heterogeneity. Recurrent attrition was analysed with an MLM with clusters at the individual and HH levels, given ICCs *>*10%. The predictive capacities of all models were checked by calculating the mean area under the curve of the receiver operating characteristic curve over a 10-fold cross-validation.^20^ Variance inflation factors (VIFs) were computed for covariates from the adjusted models to check for multicollinearity.^21^ The model code is provided inonline supplemental file 2. 

### Descriptive study design evaluation

We examined whether sharing medical findings directly with participants affected the loss to follow-up. Periportal fibrosis (PPF) status, schistosomiasis-related referrals and schistosome infection (status and a categorical indicator of schistosome infection intensity) at each timepoint were examined against attrition at a later timepoint. As exposures (schistosome infection) and outcomes (PPF) of the cohort were expected to change over time, we assessed whether there was any loss in variation of these variables by comparing baseline values of attrited versus non-attrited participants. The χ^2^ tests were used with Fisher’s exact test if sample sizes were less than five. Detailed definitions are provided in online supplemental file 1. For logistical considerations, we examined whether the study working days within villages included a Sunday. Sundays were days that most participants attended church. For each year, the overall attrition in village sampling units that included these study days was compared with village sampling units without these study days.

### Patient and public involvement

Community members were engaged early during the planning phase of the SchistoTrack cohort through local meetings with district health officials, VHTs, government nurses, community leaders, and community members. Community representatives contributed to participant recruitment and advised on optimal follow-up methods, types of compensation, and provision of clinical findings. They also played a role in the recruitment process, where VHTs facilitated the identification and mobilisation of selected HHs. Adult study participants provided direct feedback on reasons and strategies for reducing attrition.

## Results

### Attrition and rejoining metrics

Among the participants recruited at baseline, the percentage of participants lost at any follow-up timepoint was 47.4% (1341/2828), excluding deaths. Inclusion of the short-term drug-efficacy timepoint of March 2022 negligibly increased the overall attrition to 48.4% (1369/2828), which henceforth is not analysed. There were 13.2% (183/1383) of HHs with two clinical participants where both participants missed at least one timepoint together. Overall attrition ranged from 32.9% to 72.5% (median 48.1%, IQR 38.8%–54.3%) across the 38 villages sampled at baseline. Overall attrition trends across study districts were similar; Pakwach, Buliisa and Mayuge had attrition of 45% (422/938), 50.9% (478/939) and 46.4% (441/951) of study participants with at least one loss to follow-up, respectively (*χ*^2^=7.2, p=0.027).

**Figure 2:**
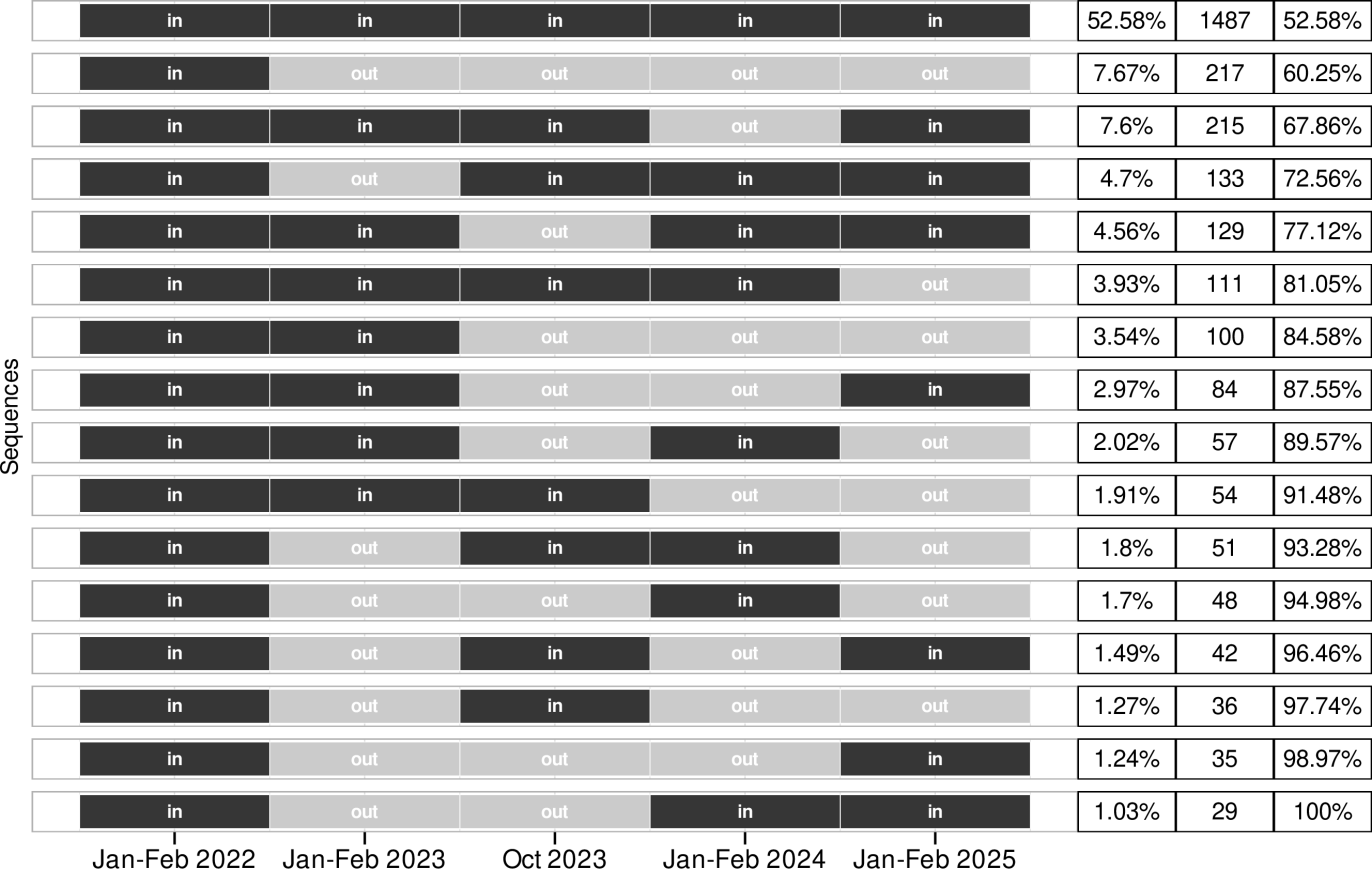
Attendance sequences. Sequences of participant attendance status are shown during the main study timepoints for the 2828 individuals recruited in 2022, excluding deaths. Each row represents a distinct pattern of attendance (‘in’) or absence (‘out’) across the five timepoints from 2022 to 2025. Sequences that include at least one instance of a participant returning (‘in’) after a period of absence (‘out’) are categorised as rejoiners.

There were 2% (57/2885) of baseline participants who died within the follow-up years, with the latest update completed in January–February 2025. For the participants recruited at baseline, the cumulative rates of attrition (of at least one loss to follow-up that included deaths) were 21.8% (629/2885) in January–February 2023, 33.3% (961/2885) in October 2023, 40.7% (1175/2885) in January–February 2024 and 48.5% (1398/2885) in January–February 2025. Only 7.7% (217/2828) of participants recruited at baseline did not return to attend any major follow-up timepoints (figure 2), and when including deaths, then only 9.5% (274/2885) of participants overall were lost.

54.1% (725/1341) of participants (excluding deaths) lost to any follow-up later rejoined the study. The percentage of attriters from January–February 2023, October 2023 and January–February 2024, who rejoined the study at the subsequent timepoint, was 40.4% (239/591), 37.4% (252/674) and 37.6% (263/699), respectively (figure 2). 26.6% (753/2828) of participants were lost to follow-up at least twice, and 15.4% (436/2828) at least thrice. 45.4% (83/183) of HHs where both individuals were lost to follow-up at the same timepoint later rejoined the study together. The range of rejoining across the 38 baseline villages was 32.4%–78.8% (median 54.6%, IQR 46.5%–63.2%). The percentage of participants who rejoined after an attrition event was 57.8% (244/422) in Pakwach, 53.6% (256/478) in Buliisa and 51% (225/441) in Mayuge (*χ*^2^=4.1, p=0.129).

**Figure 3:**
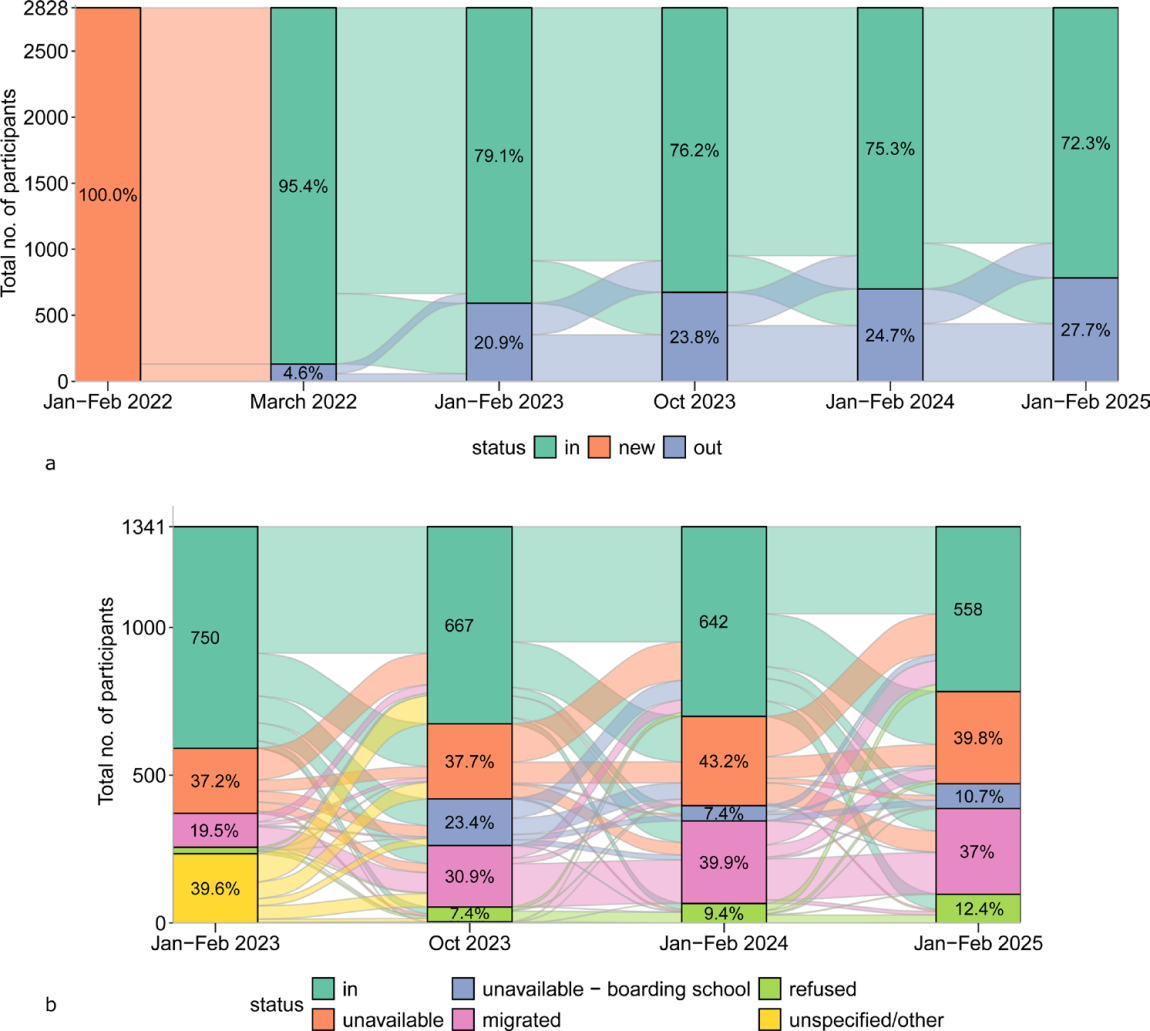
Study participation and attrition reasons across timepoints. (**a**) Study participation across all timepoints. Participant flows in and out of the study, including the March 2022 timepoint (n=2828). (**b**) Study participation at major follow-up timepoints. Participant attendance at the main follow-up timepoints (n=1341), along with reasons for attrition (n=591 in January–February 2023, n=674 in October 2023, n=699 in January–February 2024 and n=783 in January–February 2025). 3.7% (22/591) refused to participate in January–February 2023, and 0.6% (4/674) had ‘other/unspecified’ reason for attrition in October 2023. 1.3% (9/674) in October 2023, 1% (7/699) in January–February 2024 and 2.2% (17/783) in January–February 2025 were unavailable due to sickness.

Looking at any attrition at specific follow-up timepoints (excluding deaths), attrition in January–February 2023, October 2023, January–February 2024 and January–February 2025 was 20.9% (591/2828), 23.8% (674/2828), 24.7% (699/2828) and 27.7% (783/2828), respectively (figure 3a). Attrition at the previous timepoint was moderately correlated with attrition at the following timepoint (online supplemental figure S1). The median and range of attrition across the 38 baseline villages were similar across timepoints. Attrition across villages ranged from 6.2% to 35.1% (median 21.5%, IQR 16.3%–28.2%) in January–February 2023, from 10% to 41% (median 21.9%, IQR 18.9%–28.5%) in October 2023, from 10.1% to 41.6% (median 24.7%, IQR 19.4%–29%) in January–February 2024 and from 12.8% to 55% (median 26.6%, IQR 21.5%–33.6%) in January–February 2025. When looking by district and timepoint, attrition was lowest in Pakwach as compared with the other study districts, but only in January–February 2023 and January–February 2024; otherwise, attrition was similar across the districts (online supplemental table S1). Most attriters had their first attrition event in January–February 2023 (44.1%, 591/1341). Only 24% (322/1341), 15.9% (213/1341) and 16% (215/1341) of attriters had their first attrition event in October 2023, January–February 2024 and January–February 2025, respectively.

### Attrition reasons

Participant flows in and out of the study, with the reason for attrition by follow-up is presented in figure 3b. The most common reason at each timepoint was unavailability during the two clinical examination days, such as work commitments, school obligations or caretaking responsibilities, followed by migrations, with refusals, general sickness and other reasons being the least common. For example, among participants who were ever lost to follow-up, only 11.9% (160/1341) were due to refusals; among participants who refused, only 51.9% refused to participate at more than one major follow-up. Fishermen or fishmongers were not more likely to be lost to follow-up due to migration as compared with other occupations (online supplemental table S2). Apart from the seasonal timepoint, children who were enrolled in primary school were more likely to have a reason of unavailability due to being sent to boarding school (online supplemental table S2).

### Correlates of overall and recurrent attrition

**Figure 4:**
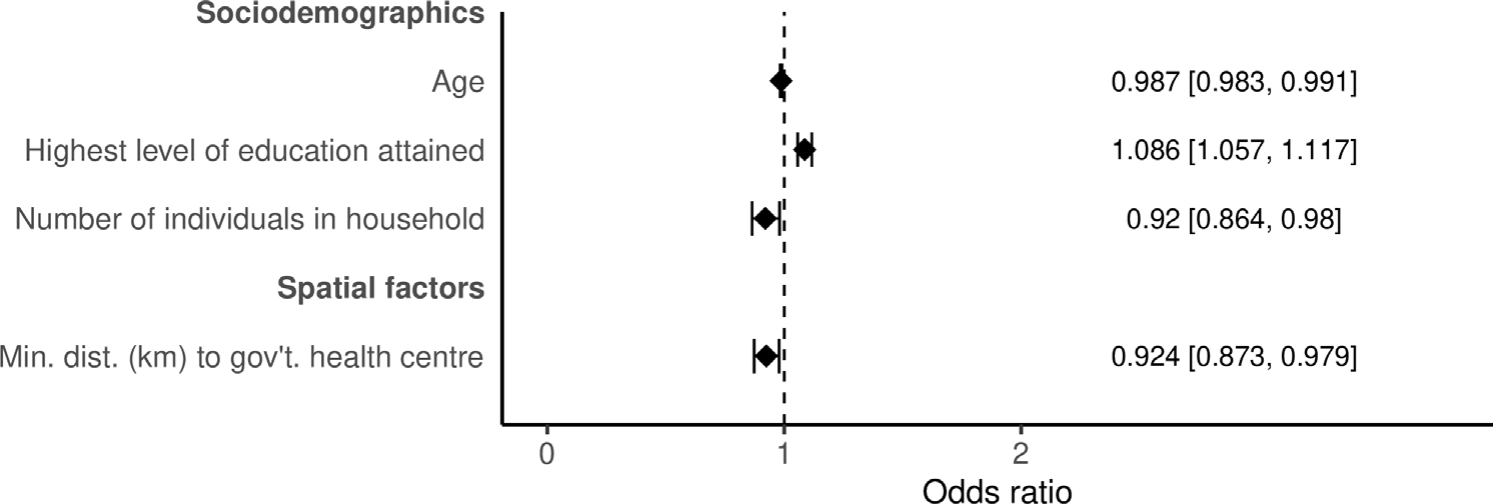
Overall attrition model. Logistic regression model for overall attrition of study participants (n=2828) with 95% CIs calculated using household-level clustered SEs (number of household clusters=1445). Variance inflation factors *<*10 for all variables. Area under the curve for 10-fold cross-validation was 0.59. Results marked with a black diamond were significant, and those marked in grey were non-significant.

The characteristics of study participants are summarised in table 1. Figure 4 shows the selected covariates against overall attrition. With each 1-year increase in age, participants were 1.3% less likely to have an attrition event (OR 0.987, 95% CI 0.983 to 0.991). Higher levels of educational attainment were positively associated with attrition (8.6%; OR 1.086, 95% CI 1.057 to 1.117). Older age was moderately positively correlated with higher educational attainment (Spearman’s ρ=0.336, p<0.001), though not collinear (VIF<10). Individuals belonging to larger HHs or homes that were in closer spatial proximity to government health centres were less likely to be lost to follow-up. Each one-member increase in HH size was correlated with an 8% lower likelihood of having an attrition event (OR 0.920, 95% CI 0.864 to 0.980). Every 1 km closer the home of a participant was to a government health centre, they were 7.6% less likely to have an attrition event (OR 0.924, 95% CI 0.873 to 0.979). Only the effect of distance to government health centres was lost when looking at potential true dropouts (online supplemental figure S2), rejoiners (online supplemental figure S3) and by any attrition at each timepoint in the study, including the seasonal timepoint (online supplemental figures S4–S7). The residuals of the logistic regression models for different time points were strongly correlated (online supplemental figure S8). Summaries of participant characteristics by each major follow-up are presented in online supplemental tables S3–S6.

**Table 1:** Participant characteristics by attrition status

	Overall	Attrited	Non-attrited	Died
	(n=2885)	(n=1341)	(n=1487)	(n=57)
**Sociodemographics**				
Age				
Mean (SD)	24.8 (18.1)	22.8 (16.4)	26.0 (19.0)	42.1 (21.9)
Sex—female	1582 (54.8%)	723 (53.9%)	835 (56.2%)	24 (42.1%)
Tribe				
Alur	1580 (54.8%)	728 (54.3%)	815 (54.8%)	37 (64.9%)
Bagungu	269 (9.3%)	138 (10.3%)	123 (8.3%)	8 (14.0%)
Musoga	466 (16.2%)	221 (16.5%)	239 (16.1%)	6 (10.5%)
Other	570 (19.8%)	254 (18.9%)	310 (20.8%)	6 (10.5%)
Majority religion	2082 (72.2%)	954 (71.1%)	1083 (72.8%)	45 (78.9%)
Highest level of education attained				
Mean (SD)	3.45 (2.82)	3.72 (2.83)	3.21 (2.80)	3.35 (2.88)
Enrolment in primary school (for children)	734 (25.4%)	375 (28.0%)	354 (23.8%)	5 (8.8%)
Occupation				
Farmer	518 (18.0%)	198 (14.8%)	310 (20.8%)	10 (17.5%)
Fisherman	251 (8.7%)	127 (9.5%)	111 (7.5%)	13 (22.8%)
Fishmonger	109 (3.8%)	43 (3.2%)	65 (4.4%)	1 (1.8%)
None/Other	2007 (69.6%)	973 (72.6%)	1001 (67.3%)	33 (57.9%)
Home quality score				
Mean (SD)	6.35 (3.55)	6.49 (3.62)	6.28 (3.49)	5.26 (3.28)
Household social status*	194 (6.7%)	89 (6.6%)	103 (6.9%)	2 (3.5%)
Number of individuals in household				
Mean (SD)	3.63 (1.38)	3.55 (1.26)	3.71 (1.49)	3.26 (1.28)
Deaths in household (past 3 years)	188 (6.5%)	74 (5.5%)	109 (7.3%)	5 (8.8%)
Years household has lived in village				
Mean (SD)	19.0 (15.0)	18.0 (14.6)	19.7 (15.1)	21.9 (18.3)
Home owned	2441 (84.6%)	1103 (82.3%)	1285 (86.4%)	53 (93.0%)
Number of rooms				
Mean (SD	2.17 (1.23)	2.14 (1.22)	2.20 (1.24)	1.91 (0.931)
**Water, sanitation and hygiene (WASH)**				
Improved drinking water source	1686 (58.4%)	774 (57.7%)	881 (59.2%)	31 (54.4%)
Household treats drinking water	654 (22.7%)	300 (22.4%)	339 (22.8%)	15 (26.3%)
Improved sanitation facility in home	1766 (61.2%)	802 (59.8%)	928 (62.4%)	36 (63.2%)
Basic hygiene facility in home	314 (10.9%)	129 (9.6%)	181 (12.2%)	4 (7%)
**Spatial factors**				
Minimum distance (km) to drug shop				
Mean (SD	1.04 (1.28)	1.00 (1.29)	1.07 (1.27)	1.24 (1.31)
Minimum distance (km) to government health centre				
Mean (SD	2.54 (1.53)	2.44 (1.48)	2.64 (1.58)	2.16 (1.28)
District				
Buliisa	963 (33.4%)	478 (35.6%)	461 (31.0%)	24 (42.1%)
Mayuge	963 (33.4%)	441 (32.9%)	510 (34.3%)	12 (21.1%)
Pakwach	959 (33.2%)	422 (31.5%)	516 (34.7%)	21 (36.8%)

Summary of participant characteristics overall and stratified by attrition status (attrited, non-attrited and died).

* Household social status indicates whether one or more members of the participant's household held a position in the local administration.

Covariate significance, directionality and effect size remained as observed for overall attrition when re-examined against the total number of attrition events (online supplemental figure S9) and recurrent attrition (online supplemental figure S10). Summary participant characteristics by the number of attrition events are presented in online supplemental table S7. For recurrent attrition, ICCs from a two-level empty multilevel model were 0.163 and 0.422 for individuals and HHs, respectively. The village-level variance (ICC) was effectively 0, and the model flagged this as a singular fit. The unexplained heterogeneity in the adjusted model (adjusted ICCs) was 0.143 for individuals and 0.425 for HHs.

### Study design evaluation

Online supplemental table S8 shows aspects of study design evaluation, including influences on attrition or attrition affecting variation in exposures and outcomes of the study focus. The return of medical findings at each timepoint did not influence study attendance at a later timepoint. Knowledge of PPF status did not influence attrition. No consistent associations of infection across timepoints with attrition were found. Only *Schistosoma mansoni* infection from January–February 2023 was unreliably associated with attrition in January–February 2024, as infection status was borderline significant (p=0.0424) and infection intensity was insignificant (p*>*0.05). Individuals requiring schistosomiasis-related medical referrals were not more likely to be lost at the next follow-up when compared with individuals who did not need a referral. Baseline values of PPF and schistosome infection status and intensity did not differ between participants lost to follow-up when looking at any attrition event with (n=2885; PPF: χ^2^=1.8, p=0.174; schistosome infection status: χ^2^=3.4, p=0.063) or without deaths (n=2828; PPF: χ^2^=0.2, p=0.653; schistosome infection status: χ^2^=3.2, p=0.074) or when comparing participants who only participated in the baseline assessments to participants who rejoined (n=942; PPF: χ^2^=0.6, p=0.438; schistosome infection status: χ^2^=0.02, p=0.897). Concerning study logistics, conducting clinical examinations on Sundays (church-going days) showed no consistent relationship with attrition (online supplemental table S9).

### Influence of study expansion

The study expansions and associated participant flows, including new recruits, are shown in figure 1 and online supplemental figure S11. Characteristics of new recruits at each timepoint are provided in online supplemental table S10. Attrition reasons of new recruits were similar to baseline recruits, except there was less attrition because of children going to boarding school (online supplemental figure S12). After study expansions, more than 3120 participants were seen in three of the follow-ups, with only the seasonal timepoint having fewer participants. A total of 3224, 2935, 3384 and 3169 participants were seen in January–February 2023, October 2023, January–February 2024 and January–February 2025, respectively. The percentage of village sampling units that had at least 65 participants was 45.8% (22/48) in January–February 2023, 20.8% (10/48) in October 2023, 87.5% (42/48) in January–February 2024 and 60.4% (29/48) in January–February 2025.

### Influence of community engagement

Following the change in participant incentives to soap in January–February 2023, there was a minor absolute difference (3.82% increase) in overall attrition when January–February 2023 was compared with January–February 2024 among the baseline (2828) study participants who would have received the original cash incentive (*χ*^2^=382.5, p*<*0.001), not accounting for the 37.6% of rejoiners in January–February 2025. Concerning active strategies to reduce attrition, there was complete data on the exact number of adult participants who attended community engagement forums in December 2023 (where attrition reasons and retention strategies were discussed) from 97.9% (47/48) of village sampling units. One of the key inputs was more advanced notice on the exact days of the study visits to help VHTs further mobilise the community and support for megaphones, which was implemented. Within the 47 village sampling units, the number of participating adults was strongly positively correlated with the number of participants at the next (January–February 2024) timepoint (Spearman’s ρ=0.629, p*<*0.001), excluding new recruits from January–February 2024. When broken down by exact village, there was no significant difference in the number of attendees when attendance in January–February 2023 was compared with attendance in January–February 2024, excluding new recruits (paired t-test, t=0.95, p=0.349).

## Discussion

Participant attrition in community-based cohorts in SSA has not been extensively explored outside of specialist populations, such as those for PLHIV or TB studies. Our study examines attrition from a general population cohort. We tracked 2885 individuals aged 5–92 over 4 years within the community-based cohort, SchistoTrack, and evaluated study expansions on the full cohort of 4321 participants. The study population represented diverse disease histories, including healthy individuals, a wide range of socioeconomic statuses and demographic characteristics, differing levels of access to healthcare, distinct geographies and varying levels of human mobility. Here we show the complexity of attrition metrics, challenge assumptions of hard-to-reach populations and provide a comprehensive framework for how future cohort studies can understand and manage attrition. The detailed set of attrition metrics provided can guide the choice of sample sizes needed for the design of future community-based cohorts in rural SSA.

The concept of attrition is inherently complex, especially in populations where individuals may rejoin the study after dropping out. Traditional views of attrition as a definitive dropout event do not capture the dynamics within rural communities. Although nearly half (47.4%) of participants were lost to follow-up at least once over 4 years, and indicators of stability and settlement, including HH size and older age, were significant in the models as seen elsewhere,^12^ attrition, as traditionally understood as a true drop-out event, was incredibly low. Only 7.7% of participants were present at baseline alone, and that total rose to only 9.5% when deaths were included for all out-of-study participants. Attrition was relatively stable at each follow-up, ranging from 20.9% to 27.7%. Conservatively, only considering sequences of individuals who were in and out of the study as opposed to in for several timepoints then out, over 54% of attriters rejoined. Although there was some time dependence (positive correlation structure) between attrition events, there was no clear trend of past attrition leading to future attrition. This finding supports the use of models with less stringent assumptions on time, such as the MLMs applied here, for examining recurrent/longitudinal attrition. Importantly, there remained some heterogeneity at the individual level (14%) and a substantial amount of unexplained heterogeneity at the HH level (42%) in all models despite detailed covariates. Instead of focusing on a long list of exclusion criteria, future studies should aim to incorporate random loss into the sampling design. Most importantly, high levels of rejoining and the lack of predictive capacity of the stability indicators underscore the need to carefully consider the minimal residency requirements when using participant or HH stability as a study inclusion criterion. HHs recruited within SchistoTrack were only required to have individuals who spent at least 6 months of the year within their village.

Our study challenges assumptions about hard-to-reach populations. Although fishing communities are often considered difficult to track due to their mobility,^22 23^ our cohort included fishing populations, and being a fisherman or fishmonger was never a discriminatory factor for predicting an attrition event, either overall or by season, the number of attrition events, recurrent attrition or differentiating between rejoiners and potential true dropouts. Individuals involved with fishing or fishmongering were not more likely to have migrated at any timepoint in the study when compared with other or no occupations. This result suggests that mobility and migration were distinct concepts, further supported by the finding that fishermen/fishmongers were just as likely to rejoin the study as any other occupation. Despite high cross-border movements with the Democratic Republic of Congo,^24^ attrition and rejoining were similar across Eastern and Western Uganda. Mobility might have been driven by varying enforcement of fishing regulations,^25^ shared tribes or HHs (with multiple spouses/homes) across villages and districts. In contrast, migration might be driven by the significant effect of higher educational attainment and individuals leaving rural areas in search of opportunities in urban centres.^26^ These differences between mobility and migration, coupled with effective community engagement, may explain why mobile individuals were likely to rejoin the study. Our study perhaps highlights a temporary form of migration related to education, where we found that enrolled school children were more likely to be unavailable during annual timepoints due to being sent to boarding school. This reason for attrition was mitigated in later timepoints through better communication of study logistics and timings of village visits. These findings indicate that mobile populations (whether fisherfolk or border communities), typically perceived as challenging to engage, may not require the stringent exclusion criteria, profiling for added community engagement or incentives, or oversampling in study designs to mitigate attrition.

Community engagement and tailored incentives may influence attrition by informing how best to respond to participant needs or offsetting opportunity costs that participants may encounter when foregoing work or other activities to participate in a study.^27^ For incentives, there was a shift in SchistoTrack from 10 000 UGX to large HH-size bars of soap. Participants were made aware of the incentive before each timepoint during sensitisation activities and participant mobilisation. The shift from in-cash to in-kind incentives was positively correlated with a slight increase in attrition the following year. However, the increase (*<*4%) was within the range of uncertainty of attrition expected year-to-year. Additionally, community feedback from forums conducted in December 2023 supported that the change in the type of incentive did not influence attendance. This finding is further supported by the stable attrition observed across the timepoints. We hosted community-based forums with adult study participants to share study findings and elicit feedback on study design and retention strategies. Within villages, adult attendance at the community-based forums was positively correlated with adult attendance at the following timepoint. Further work is needed to evaluate whether hosting forums decreases attrition (and beyond adults) and to identify whether or not villages with participants who have a higher likelihood of attending study timepoints are also more likely to attend the forums. Our findings suggest that our incentives were adequate and the type of incentive was irrelevant in the context of effective community engagement. HIV cohorts^28^ also have shown that low attrition rates were maintained by focusing on community interests in meetings and activities, along with adjustments to study logistics such as team working hours. Concerning logistics, we found no consistent effects on attrition when Sunday was included as a working day. Hence, there is a complex interplay of community engagement, incentives and logistics, where community engagement is likely to be most important for successful participant retention.

Our study design included not only the communication of medical findings but also the provision of medical referrals and transport for urgent care. This approach may be one reason for the few refusals and high rejoinings, given the role of the study in facilitating participant care continuity.^29^ Although not formally evaluated here, the provision of standard medical care and transportation reimbursement has been shown to reduce attrition.^10^ The provision of individual medical results from research studies within large-scale cohorts or biobanks is uncommon.^30^ Yet, in extremely poor settings such as the context of SchistoTrack, where care can be limited and the study may be the key source of healthcare engagement for the individual, we believed it is an ethical imperative to share such findings with the study participants and local health workers.^29^ We found no evidence that sharing such findings will deter (or potentially encourage if the individual believes they are to receive some special care, conveying a therapeutic misconception^31^ from participating in the study. Consistently, we showed that individuals belonging to homes that were in closer physical proximity to government health centres were less likely to be lost to follow-up. This result further supports the lack of therapeutic misconception because if present, then participants who may have reduced access to care might be expected to be more likely to participate in the study. By sharing medical findings, there was no apparent introduction of information bias. We did not lose any variation in key study variables such as schistosome infection status/intensity and PPF. Our experience within SchistoTrack demonstrates that understanding the study population, engaging them in meaningful dialogue and responding to their needs is essential for supporting study participation and maintaining rigorous cohort designs.

The strengths of our study lie in its large sample, detailed covariates and length of follow-up. Limitations include the lack of formal evaluation of community engagement strategies, no qualitative assessments from participants on the sharing of medical results and potential limited generalisability outside of community-based settings or rural SSA. Future research is needed to compare forums to other means of eliciting feedback from communities through clustered randomised controlled trials that could be nested within ongoing cohorts. Focus groups or semistructured individual questionnaires may be explored to understand how sharing medical findings with participants influences health-seeking behaviours or their direct perception of the role of the study in providing care. Finally, our framework requires validation in clinical settings, urban locations and other countries in SSA or elsewhere.

## Conclusion

Attrition within community-based cohorts in SSA is complex yet manageable with effective community engagement and suitable metrics. We provide a starting set of statistics for other researchers to design recruitment and sampling strategies for community-based cohorts, as well as analytical and conceptual frameworks for evaluating attrition in a manner that assesses effects on study outcomes and prioritises participant needs. If replicated in other studies, the attrition findings and retention strategies from SchistoTrack could help to construct guidelines for the robust design of large-scale community-based cohorts in SSA.

## Supplementary material

10.1136/bmjph-2024-002151 online supplemental file 1

10.1136/bmjph-2024-002151 online supplemental file 2

## Data Availability

Individual participant data is not available due to the identifiable nature of the participant characteristics and ongoing nature of the cohort. Extensive metadata relevant to the study are included in the article or uploaded as supplementary information. Model code is provided as supplementary material.
