# Supplementary material for: Design and maintenance of community-based cohorts in sub-Saharan Africa: a longitudinal evaluation of participant attrition in SchistoTrack
Source: BMJ Public Health. 2025 Aug 10;3(2):e002151. doi: 10.1136/bmjph-2024-002151 (PMC12336594; doi:10.1136/bmjph-2024-002151)
Supplement: online supplemental file 1 [file bmjph-3-2-s001.pdf]

Supplementary information

S1 Supplementary methods

S1.1 Covariate definitions

Age was recorded as a continuous variable in years at the time of recruitment. Sex was a binary variable coded as male or female with male being the reference category. Tribe was categorised for the three majority tribes of Alur, Musoga, and Bagungu, with the reference category being all other tribes. Majority religion was a binary variable indicating whether the participant belonged to the predominant religion in their village, with not belonging as the reference category. Enrolment in primary school was a binary variable indicating whether a participant aged below 18 years was enrolled in primary school at the time of recruitment, with non-enrolment being the reference category. The highest level of education attained was an ordinal variable ranging from 0 to 14, with 0 corresponding to no education; 1-7 to primary level education; 8-12 to senior level education; 13 to diploma, certificate, or some university education, which are considered a form of advanced education in Uganda; and 14 to completed university education [1]. The reference level was 0, indicating no level of primary education was completed. Occupation was categorised into farmer, fisherman, and fishmonger with no occupations and other occupations as one reference category. The occupation variable was curated to capture the behaviour of populations at low and high risks of attrition based on assumed differences in mobility given fishermen move to areas with the best catches and farmers have designated set land for which to use for subsistence.

The home quality score variable was a continuous variable generated by ranking the materials of the roof, walls, and floor for each HH on a scale from 1 to 4, and then summing these ranks. We followed Chami et al. [1] for the ranking of materials, with the order from lowest to highest quality as follows: for the roof, grass or papyrus, sticks, plastic, and metal; for the walls, mud and sticks, plastic, metal, and bricks or cement; and for the floor, mud, plastic, wood planks, and brick or cement. HH social status was a binary variable indicating whether one or more members of the HH held a position in the local administration, with no members holding an administrative position as the reference category. The number of individuals in the HH, indicating HH size, was recorded as a count. Deaths in the HH in the three years before recruitment were coded as a binary variable, with the reference category indicating the absence of deaths in the HH. The number of years the HH has lived in the village, suggestive of HH stability, was recorded as a continuous variable. Home ownership was a binary variable, with the reference level being that the HH head did not own their home and rented the home. The number of rooms in the home was recorded as a count, reflecting HH density and living conditions as well as possible wealth.

HH-level variables related to water, sanitation, and hygiene (WASH) were considered in the analysis as preventive health measures potentially influencing participation in the study. An improved drinking water source [2] was included as a binary variable, with sources such as a protected well or spring, borehole, village tap, or rainwater classified as improved, while other sources served as the

reference category. HH water treatment [3] was included as a binary variable, with no water treatment or efforts to purify or make the water safe for consumption after collection as the reference value. Methods of water treatment considered included boiling, adding bleach or chlorine, straining through a cloth, using a water filter, solar disinfection, washing the jerrycan used for storing water with soap, and letting the water stand and settle. Having an improved sanitation facility [2], such as a flush toilet or a covered latrine with privacy, was another binary indicator, with the absence of such a facility as the reference level. Additionally, having provisions for washing hands with water and soap was included as a binary variable, with the lack of such provisions as the reference.

Regarding health infrastructure, study participants have access to healthcare services through a combination of private drug shops and government health centres of varying levels (e.g., Health Centre II (HCII), Health Centre III (HCIII)) within their respective districts. HCIIs offer preventive, promotive, outpatient curative, outreach, and emergency care, typically serving around 5,000 people. HCIIIs provide a broader range of services, including maternity care, inpatient services and basic laboratory diagnostics, and serve populations of approximately 20,000. In the study catchment areas, government health infrastructure includes three health centres in Pakwach (two HCIIIs and one HCII), one HCIII in Buliisa, and four in Mayuge (one HCIII and three HCIIs). The spatial factors of distances to drug shops and health centres were calculated using the `raster` package in R [4], as the shortest distance between the waypoint coordinates of the drug shops and health centres, collected by surveyors in each district, and the waypoint coordinates of each HH in the respective district. HHs were iteratively compared to each drug shop and health center within their district to determine the closest distances. The positional accuracy of the locations was constrained by the average waypoint accuracy of 5 metres for the drug shops and health centres, and 10 metres for the HH locations, as recorded in the ODK surveys. These spatial factors were considered to account for variations in access to health services and infrastructure, which could influence participation in the study.

S1.2 Medical variable definitions

PPF was diagnosed with point-of-care ultrasound following the Niamey Protocol [5] as described in detail elsewhere [6]. All detected patterns of PPF were documented, and the most severe pattern observed for each participant was used to create a binary outcome variable. In this classification, any participant with a pattern in the C-F range was categorised as having PPF, while patterns in the A-B range were classified as no PPF. Schistosomiasis-related referrals were mainly due to low haemoglobin counts, severe liver fibrotic disease from ultrasound scans, hepatitis-like livers from ultrasound scans, nurse-determined urgent needs for blood transfusion, self-reported history of vomiting blood, portal hypertension, splenomegaly, hepatomegaly, liver cirrhosis, need for praziquantel treatment, ascites, and observed liver masses from ultrasound as unclear whether schistosomiasis-related or due to other causes. Schistosome infection status was ascertained using Kato-Katz (KK) microscopy [7] in which two thick smear slides were prepared from a single stool sample, and the two slides were examined by two different technicians. The results from the slides were then averaged and multiplied by 24 to estimate the number of eggs per gram (EPG) of faeces. A positive KK infection status was determined when EPG>=1. For infection intensity, WHO categories [8] were applied, where the classifications were no infection (0 EPG), low (1-99 EPG), moderate (100-399 EPG), and heavy (400+ EPG).

S2 Supplementary tables

Table S1: **Attrition by district.** Attrition in study districts at major study timepoints [1].

District	Jan-Feb 2023	Oct 2023	Jan-Feb 2024	Jan-Feb 2025
Pakwach	18.6% (174/938)	23.2% (218/938)	20.3% (190/938)	24.6% (231/938)
Buliisa	24.8% (233/939)	26.2% (246/939)	26.2% (246/939)	30.1% (283/939)
Mayuge	19.3% (184/951)	22.1% (210/951)	27.7% (263/951)	28.3% (269/951)

[1] Chi-squared test statistics and p-values for group differences: Jan–Feb 2023: $\chi^2 = 13.22$, $p = 0.001$; Oct 2023: $\chi^2 = 4.68$, $p = 0.096$; Jan–Feb 2024: $\chi^2 = 15.55$, $p < 0.001$; Jan–Feb 2025: $\chi^2 = 7.38$, $p = 0.025$.

Table S2: **Migration and school obligations.** Attrition reasons of migration among fishermen/fishmongers, and school obligations among children enrolled in primary school [1].

Timepoint	Fisherman/ Fishmonger	Reason: migrated	Reason: other/unknown	Proportion (%)	χ^2	p-value
Jan-Feb 2023	0	98	412	19.2	0.05	0.823
Jan-Feb 2023	1	17	64	21.0	0.05	0.823
Oct 2023	0	183	417	30.5	0.2	0.657
Oct 2023	1	25	49	33.8	0.2	0.657
Jan-Feb 2024	0	247	382	39.3	0.84	0.360
Jan-Feb 2024	1	32	38	45.7	0.84	0.360
Jan-Feb 2025	0	256	430	37.3	0.10	0.749
Jan-Feb 2025	1	34	63	35.1	0.10	0.749
Any attrition	0	453	718	38.7	0.10	0.747
Any attrition	1	63	107	37.1	0.10	0.747
	Enrolled in primary school	Reason: school	Reason: other/unknown			
Oct 2023	no	76	122	38.4	0.25	0.620
Oct 2023	yes	79	112	41.4	0.25	0.620
Jan-Feb 2024	no	4	178	2.2	29.59	<0.001
Jan-Feb 2024	yes	46	178	20.5	29.59	<0.001
Jan-Feb 2025	no	16	191	7.7	29.82	<0.001
Jan-Feb 2025	yes	68	171	28.5	29.82	<0.001
Any attrition	no	89	255	25.9	11.79	<0.001
Any attrition	yes	143	232	38.1	11.79	<0.001

[1] In the first part of the table, which examines migration-related attrition among fishermen/fishmongers, denominators represent the number of attriters at each timepoint: 591 (Jan–Feb 2023), 674 (Oct 2023), 699 (Jan–Feb 2024), 783 (Jan–Feb 2025), and 1341 (any attrition). In the second part, which examines school-related attrition among participants under 18 enrolled in primary school, denominators refer to attriters under 18 at each timepoint: 389 (Oct 2023), 406 (Jan–Feb 2024), 446 (Jan–Feb 2025), and 719 (any attrition).

Table S3: **Participant characteristics by attrition status in Jan-Feb 2023.** Summary of participant characteristics overall and stratified by attrition status in Jan-Feb 2023.

	Overall	Attrited	Non-attrited
	(N=2828)	(N=591)	(N=2237)
SOCIODEMOGAPHICS			
Age			
Mean (SD)	24.5 (17.9)	22.4 (16.1)	25.0 (18.3)
Sex - Female	1558 (55.1%)	337 (57.0%)	1221 (54.6%)
Tribe			
Alur	1543 (54.6%)	331 (56.0%)	1212 (54.2%)
Bagungu	261 (9.2%)	66 (11.2%)	195 (8.7%)
Musoga	460 (16.3%)	97 (16.4%)	363 (16.2%)
Other	564 (19.9%)	97 (16.4%)	467 (20.9%)
Majority religion	2037 (72.0%)	412 (69.7%)	1625 (72.6%)
Highest level of education attained			
Mean (SD)	3.45 (2.82)	3.81 (2.98)	3.36 (2.77)
Enrolment in primary school (for children)	729 (25.8%)	168 (28.4%)	561 (25.1%)
Occupation			
Farmer	508 (18.0%)	75 (12.7%)	433 (19.4%)
Fisherman	238 (8.4%)	58 (9.8%)	180 (8.0%)
Fishmonger	108 (3.8%)	23 (3.9%)	85 (3.8%)
None/Other	1974 (69.8%)	435 (73.6%)	1539 (68.8%)
Home quality score			
Mean (SD)	6.38 (3.56)	6.40 (3.56)	6.37 (3.55)
HH social status	192 (6.8%)	37 (6.3%)	155 (6.9%)
Number of individuals in HH			
Mean (SD)	3.64 (1.39)	3.42 (1.16)	3.69 (1.43)
Deaths in HH (past 3 yrs)	183 (6.5%)	25 (4.2%)	158 (7.1%)
Years HH has lived in village			
Mean (SD)	18.9 (14.9)	17.0 (14.2)	19.4 (15.0)
Home owned	2388 (84.4%)	466 (78.8%)	1922 (85.9%)
Number of rooms			
Mean (SD)	2.17 (1.23)	2.04 (1.11)	2.21 (1.26)
WATER, SANITATION AND HYGIENE (WASH)			
Improved drinking water source	1655 (58.5%)	357 (60.4%)	1298 (58.0%)
HH treats drinking water	639 (22.6%)	127 (21.5%)	512 (22.9%)
Improved sanitation facility in home	1730 (61.2%)	325 (55.0%)	1405 (62.8%)
Basic hygiene facility in home	310 (11.0%)	44 (7.4%)	266 (11.9%)
SPATIAL FACTORS			
Min. dist. (km) to drug shop			
Mean (SD)	1.04 (1.28)	1.02 (1.34)	1.04 (1.27)
Min. dist. (km) to gov't. health centre			
Mean (SD)	2.54 (1.53)	2.39 (1.39)	2.58 (1.57)
District			
Buliisa	939 (33.2%)	233 (39.4%)	706 (31.6%)
Mayuge	951 (33.6%)	184 (31.1%)	767 (34.3%)
Pakwach	938 (33.2%)	174 (29.4%)	764 (34.2%)

Table S4: **Participant characteristics by attrition status in Oct 2023.** Summary of participant characteristics overall and stratified by attrition status in Oct 2023.

	Overall	Attrited	Non-attrited
	(N=2828)	(N=674)	(N=2154)
SOCIODEMOGAPHICS			
Age			
Mean (SD)	24.5 (17.9)	21.0 (15.2)	25.6 (18.5)
Sex - Female	1558 (55.1%)	382 (56.7%)	1176 (54.6%)
Tribe			
Alur	1543 (54.6%)	384 (57.0%)	1159 (53.8%)
Bagungu	261 (9.2%)	66 (9.8%)	195 (9.1%)
Musoga	460 (16.3%)	107 (15.9%)	353 (16.4%)
Other	564 (19.9%)	117 (17.4%)	447 (20.8%)
Majority religion	2037 (72.0%)	484 (71.8%)	1553 (72.1%)
Highest level of education attained			
Mean (SD)	3.45 (2.82)	3.74 (2.86)	3.36 (2.81)
Enrolment in primary school (for children)	729 (25.8%)	191 (28.3%)	538 (25.0%)
Occupation			
Farmer	508 (18.0%)	90 (13.4%)	418 (19.4%)
Fisherman	238 (8.4%)	52 (7.7%)	186 (8.6%)
Fishmonger	108 (3.8%)	22 (3.3%)	86 (4.0%)
None/Other	1974 (69.8%)	510 (75.7%)	1464 (68.0%)
Home quality score			
Mean (SD)	6.38 (3.56)	6.40 (3.64)	6.37 (3.53)
HH social status	192 (6.8%)	45 (6.7%)	147 (6.8%)
Number of individuals in HH			
Mean (SD)	3.64 (1.39)	3.50 (1.22)	3.68 (1.43)
Deaths in HH (past 3 yrs)	183 (6.5%)	36 (5.3%)	147 (6.8%)
Years HH has lived in village			
Mean (SD)	18.9 (14.9)	17.8 (15.1)	19.3 (14.8)
Home owned	2388 (84.4%)	548 (81.3%)	1840 (85.4%)
Number of rooms			
Mean (SD)	2.17 (1.23)	2.13 (1.32)	2.19 (1.20)
WATER, SANITATION AND HYGIENE (WASH)			
Improved drinking water source	1655 (58.5%)	387 (57.4%)	1268 (58.9%)
HH treats drinking water	639 (22.6%)	154 (22.8%)	485 (22.5%)
Improved sanitation facility in home	1730 (61.2%)	401 (59.5%)	1329 (61.7%)
Basic hygiene facility in home	310 (11.0%)	59 (8.8%)	251 (11.7%)
SPATIAL FACTORS			
Min. dist. (km) to drug shop			
Mean (SD)	1.04 (1.28)	1.04 (1.35)	1.04 (1.26)
Min. dist. (km) to gov't. health centre			
Mean (SD)	2.54 (1.53)	2.39 (1.47)	2.59 (1.55)
District			
Buliisa	939 (33.2%)	246 (36.5%)	693 (32.2%)
Mayuge	951 (33.6%)	210 (31.2%)	741 (34.4%)
Pakwach	938 (33.2%)	218 (32.3%)	720 (33.4%)

Table S5: **Participant characteristics by attrition status in Jan-Feb 2024.** Summary of participant characteristics overall and stratified by attrition status in Jan-Feb 2024.

	Overall	Attrited	Non-attrited
	(N=2828)	(N=699)	(N=2129)
SOCIODEMOGAPHICS			
Age			
Mean (SD)	24.5 (17.9)	21.2 (15.2)	25.6 (18.5)
Sex - Female	1558 (55.1%)	385 (55.1%)	1173 (55.1%)
Tribe			
Alur	1543 (54.6%)	353 (50.5%)	1190 (55.9%)
Bagungu	261 (9.2%)	67 (9.6%)	194 (9.1%)
Musoga	460 (16.3%)	132 (18.9%)	328 (15.4%)
Other	564 (19.9%)	147 (21.0%)	417 (19.6%)
Majority religion	2037 (72.0%)	486 (69.5%)	1551 (72.9%)
Highest level of education attained			
Mean (SD)	3.45 (2.82)	3.65 (2.81)	3.39 (2.83)
Enrolment in primary school (for children)	729 (25.8%)	224 (32.0%)	505 (23.7%)
Occupation			
Farmer	508 (18.0%)	96 (13.7%)	412 (19.4%)
Fisherman	238 (8.4%)	49 (7.0%)	189 (8.9%)
Fishmonger	108 (3.8%)	21 (3.0%)	87 (4.1%)
None/Other	1974 (69.8%)	533 (76.3%)	1441 (67.7%)
Home quality score			
Mean (SD)	6.38 (3.56)	6.64 (3.60)	6.29 (3.54)
HH social status	192 (6.8%)	36 (5.2%)	156 (7.3%)
Number of individuals in HH			
Mean (SD)	3.64 (1.39)	3.51 (1.24)	3.68 (1.43)
Deaths in HH (past 3 yrs)	183 (6.5%)	41 (5.9%)	142 (6.7%)
Years HH has lived in village			
Mean (SD)	18.9 (14.9)	17.6 (14.5)	19.3 (15.0)
Home owned	2388 (84.4%)	568 (81.3%)	1820 (85.5%)
Number of rooms			
Mean (SD)	2.17 (1.23)	2.14 (1.21)	2.18 (1.24)
WATER, SANITATION AND HYGIENE (WASH)			
Improved drinking water source	1655 (58.5%)	398 (56.9%)	1257 (59.0%)
HH treats drinking water	639 (22.6%)	147 (21.0%)	492 (23.1%)
Improved sanitation facility in home	1730 (61.2%)	424 (60.7%)	1306 (61.3%)
Basic hygiene facility in home	310 (11.0%)	67 (9.6%)	243 (11.4%)
SPATIAL FACTORS			
Min. dist. (km) to drug shop			
Mean (SD)	1.04 (1.28)	0.927 (1.25)	1.07 (1.29)
Min. dist. (km) to gov't. health centre			
Mean (SD)	2.54 (1.53)	2.48 (1.47)	2.56 (1.55)
District			
Buliisa	939 (33.2%)	246 (35.2%)	693 (32.6%)
Mayuge	951 (33.6%)	263 (37.6%)	688 (32.3%)
Pakwach	938 (33.2%)	190 (27.2%)	748 (35.1%)

Table S6: **Participant characteristics by attrition status in Jan-Feb 2025.** Summary of participant characteristics overall and stratified by attrition status in Jan-Feb 2025.

	Overall	Attrited	Non-attrited
	(N=2828)	(N=783)	(N=2045)
SOCIODEMOGAPHICS			
Age			
Mean (SD)	24.5 (17.9)	21.5 (15.6)	25.6 (18.6)
Sex - Female	1558 (55.1%)	407 (52.0%)	1151 (56.3%)
Tribe			
Alur	1543 (54.6%)	405 (51.7%)	1138 (55.6%)
Bagungu	261 (9.2%)	78 (10.0%)	183 (8.9%)
Musoga	460 (16.3%)	134 (17.1%)	326 (15.9%)
Other	564 (19.9%)	166 (21.2%)	398 (19.5%)
Majority religion	2037 (72.0%)	550 (70.2%)	1487 (72.7%)
Highest level of education attained			
Mean (SD)	3.45 (2.82)	3.81 (2.78)	3.31 (2.83)
Enrolment in primary school (for children)	729 (25.8%)	239 (30.5%)	490 (24.0%)
Occupation			
Farmer	508 (18.0%)	96 (12.3%)	412 (20.1%)
Fisherman	238 (8.4%)	75 (9.6%)	163 (8.0%)
Fishmonger	108 (3.8%)	22 (2.8%)	86 (4.2%)
None/Other	1974 (69.8%)	590 (75.4%)	1384 (67.7%)
Home quality score			
Mean (SD)	6.38 (3.56)	6.56 (3.64)	6.31 (3.52)
HH social status	192 (6.8%)	56 (7.2%)	136 (6.7%)
Number of individuals in HH			
Mean (SD)	3.64 (1.39)	3.49 (1.19)	3.69 (1.45)
Deaths in HH (past 3 yrs)	183 (6.5%)	46 (5.9%)	137 (6.7%)
Years HH has lived in village			
Mean (SD)	18.9 (14.9)	17.7 (14.9)	19.4 (14.9)
Home owned	2388 (84.4%)	626 (79.9%)	1762 (86.2%)
Number of rooms			
Mean (SD)	2.17 (1.23)	2.08 (1.17)	2.21 (1.25)
WATER, SANITATION AND HYGIENE (WASH)			
Improved drinking water source	1655 (58.5%)	440 (56.2%)	1215 (59.4%)
HH treats drinking water	639 (22.6%)	178 (22.7%)	461 (22.5%)
Improved sanitation facility in home	1730 (61.2%)	472 (60.3%)	1258 (61.5%)
Basic hygiene facility in home	310 (11.0%)	71 (9.1%)	239 (11.7%)
SPATIAL FACTORS			
Min. dist. (km) to drug shop			
Mean (SD)	1.04 (1.28)	0.946 (1.26)	1.07 (1.29)
Min. dist. (km) to gov't. health centre			
Mean (SD)	2.54 (1.53)	2.43 (1.48)	2.59 (1.55)
District			
Buliisa	939 (33.2%)	283 (36.1%)	656 (32.1%)
Mayuge	951 (33.6%)	269 (34.4%)	682 (33.3%)
Pakwach	938 (33.2%)	231 (29.5%)	707 (34.6%)

Table S7: **Participant characteristics by number of attrition events.** Summary of participant characteristics overall and stratified by number of attrition events (0, 1, 2, 3, and 4).

	Overall	0	1	2	3	4
	(N=2828)	(N=1487)	(N=588)	(N=317)	(N=219)	(N=217)
SOCIODEMOGAPHICS						
Age						
Mean (SD)	24.5 (17.9)	26.0 (19.0)	25.2 (17.7)	22.4 (15.8)	20.7 (14.8)	18.8 (13.7)
Sex - Female	1558 (55.1%)	835 (56.2%)	303 (51.5%)	172 (54.3%)	128 (58.4%)	120 (55.3%)
Tribe						
Alur	1543 (54.6%)	815 (54.8%)	317 (53.9%)	187 (59.0%)	114 (52.1%)	110 (50.7%)
Bagungu	261 (9.2%)	123 (8.3%)	65 (11.1%)	30 (9.5%)	20 (9.1%)	23 (10.6%)
Musoga	460 (16.3%)	239 (16.1%)	91 (15.5%)	49 (15.5%)	43 (19.6%)	38 (17.5%)
Other	564 (19.9%)	310 (20.8%)	115 (19.6%)	51 (16.1%)	42 (19.2%)	46 (21.2%)
Majority religion	2037 (72.0%)	1083 (72.8%)	429 (73.0%)	222 (70.0%)	153 (69.9%)	150 (69.1%)
Highest level of education attained						
Mean (SD)	3.45 (2.82)	3.21 (2.80)	3.56 (2.82)	3.94 (2.74)	3.89 (2.91)	3.65 (2.91)
Enrolment in primary school (for children)	729 (25.8%)	354 (23.8%)	139 (23.6%)	91 (28.7%)	79 (36.1%)	66 (30.4%)
Occupation						
Farmer	508 (18.0%)	310 (20.8%)	104 (17.7%)	47 (14.8%)	29 (13.2%)	18 (8.3%)
Fisherman	238 (8.4%)	111 (7.5%)	70 (11.9%)	24 (7.6%)	16 (7.3%)	17 (7.8%)
Fishmonger	108 (3.8%)	65 (4.4%)	18 (3.1%)	8 (2.5%)	14 (6.4%)	3 (1.4%)
None/Other	1974 (69.8%)	1001 (67.3%)	396 (67.3%)	238 (75.1%)	160 (73.1%)	179 (82.5%)
Home quality score						
Mean (SD)	6.38 (3.56)	6.28 (3.49)	6.42 (3.61)	6.56 (3.69)	6.63 (3.62)	6.44 (3.56)
HH social status	192 (6.8%)	103 (6.9%)	38 (6.5%)	25 (7.9%)	18 (8.2%)	8 (3.7%)
Number of individuals in HH						
Mean (SD)	3.64 (1.39)	3.71 (1.49)	3.64 (1.37)	3.66 (1.22)	3.39 (1.03)	3.32 (1.16)
Deaths in HH (past 3 yrs)	183 (6.5%)	109 (7.3%)	33 (5.6%)	21 (6.6%)	7 (3.2%)	13 (6.0%)
Years HH has lived in village						
Mean (SD)	18.9 (14.9)	19.7 (15.1)	18.6 (14.4)	18.0 (14.6)	18.4 (15.6)	15.9 (14.1)
Home owned	2388 (84.4%)	1285 (86.4%)	502 (85.4%)	262 (82.6%)	174 (79.5%)	165 (76.0%)
Number of rooms						
Mean (SD)	2.17 (1.23)	2.20 (1.24)	2.18 (1.21)	2.18 (1.22)	2.21 (1.50)	1.90 (0.874)
WATER, SANITATION AND HYGIENE (WASH)						
Improved drinking water source	1655 (58.5%)	881 (59.2%)	339 (57.7%)	180 (56.8%)	137 (62.6%)	118 (54.4%)
HH treats drinking water	639 (22.6%)	339 (22.8%)	129 (21.9%)	80 (25.2%)	47 (21.5%)	44 (20.3%)
Improved sanitation facility in home	1730 (61.2%)	928 (62.4%)	368 (62.6%)	173 (54.6%)	136 (62.1%)	125 (57.6%)
Basic hygiene facility in home	310 (11.0%)	181 (12.2%)	62 (10.5%)	35 (11.0%)	19 (8.7%)	13 (6.0%)
SPATIAL FACTORS						
Min. dist. (km) to drug shop						
Mean (SD)	1.04 (1.28)	1.07 (1.27)	1.06 (1.28)	0.950 (1.29)	1.00 (1.32)	0.933 (1.30)
Min. dist. (km) to gov't. health centre						
Mean (SD)	2.54 (1.53)	2.64 (1.58)	2.41 (1.49)	2.64 (1.53)	2.26 (1.44)	2.40 (1.37)
District						
Buliisa	939 (33.2%)	461 (31.0%)	197 (33.5%)	114 (36.0%)	85 (38.8%)	82 (37.8%)
Mayuge	951 (33.6%)	510 (34.3%)	188 (32.0%)	95 (30.0%)	84 (38.4%)	74 (34.1%)
Pakwach	938 (33.2%)	516 (34.7%)	203 (34.5%)	108 (34.1%)	50 (22.8%)	61 (28.1%)

Table S8: **Study exposures and outcomes by later attrition status.** Summary of schistosomiasis infection, periportal fibrosis, and medical referrals among participants stratified by whether they attrited from the study at a later timpoint.

	Total	In	Out	P-value
		Jan-Feb 2023		
	(N=2791)	(N=2207)	(N=584)	
S. mansoni (Jan-Feb 2022)	1213 (43.5%)	959 (43.5%)	254 (43.5%)	1
Eggs per gram category (WHO) (Jan-Feb 2022)				0.964
No	1578 (56.5%)	1248 (56.5%)	330 (56.5%)	
High	231 (8.3%)	182 (8.2%)	49 (8.4%)	
Mild	360 (12.9%)	288 (13.0%)	72 (12.3%)	
Low	622 (22.3%)	489 (22.2%)	133 (22.8%)	
Periportal fibrosis (Jan-Feb 2022)	321 (11.5%)	255 (11.6%)	66 (11.3%)	0.922
Any schistosomiasis-related referrals (Jan-Feb 2022)	71 (2.5%)	58 (2.6%)	13 (2.2%)	0.689
		Oct 2023		
	(N=2198)	(N=1893)	(N=305)	
S. mansoni (Jan-Feb 2023)	721 (32.8%)	619 (32.7%)	102 (33.4%)	0.849
Eggs per gram category (WHO) (Jan-Feb 2023)				0.535
No	1477 (67.2%)	1274 (67.3%)	203 (66.6%)	
High	102 (4.6%)	89 (4.7%)	13 (4.3%)	
Mild	165 (7.5%)	136 (7.2%)	29 (9.5%)	
Low	454 (20.7%)	394 (20.8%)	60 (19.7%)	
Periportal fibrosis (Jan-Feb 2023)	344 (15.7%)	304 (16.1%)	40 (13.1%)	0.219
Any schistosomiasis-related referrals (Jan-Feb 2023)	5 (0.2%)	5 (0.3%)	0 (0%)	1
		Jan-Feb 2024		
	(N=2126)	(N=1859)	(N=267)	
S. mansoni (Oct 2023)	880 (41.4%)	770 (41.4%)	110 (41.2%)	0.998
Eggs per gram category (WHO) (Oct 2023)				0.999
No	1246 (58.6%)	1089 (58.6%)	157 (58.8%)	
High	184 (8.7%)	161 (8.7%)	23 (8.6%)	
Mild	220 (10.3%)	192 (10.3%)	28 (10.5%)	
Low	476 (22.4%)	417 (22.4%)	59 (22.1%)	
		Jan-Feb 2024		
	(N=2198)	(N=1847)	(N=351)	
S. mansoni (Jan-Feb 2023)	721 (32.8%)	589 (31.9%)	132 (37.6%)	0.0424
Eggs per gram category (WHO) (Jan-Feb 2023)				0.121
No	1477 (67.2%)	1258 (68.1%)	219 (62.4%)	
High	102 (4.6%)	87 (4.7%)	15 (4.3%)	
Mild	165 (7.5%)	136 (7.4%)	29 (8.3%)	
Low	454 (20.7%)	366 (19.8%)	88 (25.1%)	
Periportal fibrosis (Jan-Feb 2023)	344 (15.7%)	296 (16.0%)	48 (13.7%)	0.303
Any schistosomiasis-related referrals (Jan-Feb 2023)	5 (0.2%)	4 (0.2%)	1 (0.3%)	0.581
		Jan-Feb 2025		
	(N=2056)	(N=1742)	(N=314)	
S. mansoni (Jan-Feb 2024)	872 (42.4%)	733 (42.1%)	139 (44.3%)	0.509
Eggs per gram category (WHO) (Jan-Feb 2024)				0.76
No	1184 (57.6%)	1009 (57.9%)	175 (55.7%)	
High	210 (10.2%)	177 (10.2%)	33 (10.5%)	
Mild	234 (11.4%)	193 (11.1%)	41 (13.1%)	
Low	428 (20.8%)	363 (20.8%)	65 (20.7%)	
Periportal fibrosis (Jan-Feb 2024)	398 (19.4%)	330 (18.9%)	68 (21.7%)	0.297
Any schistosomiasis-related referrals (Jan-Feb 2024)	13 (0.6%)	11 (0.6%)	2 (0.6%)	1

Table S9: **Attrition by inclusion of Sundays in village study days.** Comparison of participant attrition rates between villages where study days included Sundays and those that did not include Sundays.

	Overall	Included Sunday	No Sunday	P-value
	(N=2828)	(N=315)	(N=2513)	
Attrition in Jan-Feb 2023	591 (20.9%)	49 (15.6%)	542 (21.6%)	0.0164
	(N=2828)	(N=79)	(N=2749)	
Attrition in Oct 2023	674 (23.8%)	15 (19.0%)	659 (24.0%)	0.373
	(N=2828)	(N=709)	(N=2119)	
Attrition in Jan-Feb 2024	699 (24.7%)	199 (28.1%)	500 (23.6%)	0.0193
	(N=2828)	(N=865)	(N=1963)	
Attrition in Jan-Feb 2025	783 (27.7%)	225 (26.0%)	558 (28.4%)	0.202

Table S10: **Participant characteristics of new recruits.** Summary of participant characteristics for all new recruits and stratified by year of recruitment.

	All new recruits	2023 new recruits	2024 new recruits
	(N=1420)	(N=954)	(N=466)
SOCIODEMOGAPHICS			
Age			
Mean (SD)	25.2 (18.3)	24.9 (17.9)	25.7 (19.1)
Sex - Female	762 (53.7%)	496 (52.0%)	266 (57.1%)
Tribe			
Alur	1050 (73.9%)	796 (83.4%)	254 (54.5%)
Bagungu	139 (9.8%)	89 (9.3%)	50 (10.7%)
Musoga	61 (4.3%)	0 (0%)	61 (13.1%)
Other	170 (12.0%)	69 (7.2%)	101 (21.7%)
Majority religion	1157 (81.5%)	834 (87.4%)	323 (69.3%)
Highest level of education attained			
Mean (SD)	3.10 (2.79)	3.02 (2.78)	3.27 (2.79)
Enrolment in primary school (for children)	109 (7.7%)	62 (6.5%)	47 (10.1%)
Occupation			
Farmer	177 (12.5%)	113 (11.8%)	64 (13.7%)
Fisherman	58 (4.1%)	37 (3.9%)	21 (4.5%)
Fishmonger	37 (2.6%)	19 (2.0%)	18 (3.9%)
None/Other	1148 (80.8%)	785 (82.3%)	363 (77.9%)
Home quality score			
Mean (SD)	4.53 (2.82)	3.81 (2.19)	6.03 (3.33)
HH social status	144 (10.1%)	92 (9.6%)	52 (11.2%)
Number of individuals in HH	3.75 (1.61)	3.55 (1.40)	4.17 (1.91)
Deaths in HH (past 3 yrs)	133 (9.4%)	105 (11.0%)	28 (6.0%)
Years HH has lived in village	22.2 (16.7)	21.3 (17.3)	24.1 (15.4)
Home owned	1287 (90.6%)	861 (90.3%)	426 (91.4%)
Number of rooms			
Mean (SD)	2.10 (1.04)	1.97 (0.992)	2.37 (1.08)
WATER, SANITATION AND HYGIENE (WASH)			
Improved drinking water source	646 (45.5%)	483 (50.6%)	163 (35.0%)
HH treats drinking water	309 (21.8%)	206 (21.6%)	103 (22.1%)
Improved sanitation facility in home	703 (49.5%)	415 (43.5%)	288 (61.8%)
Basic hygiene facility in home	50 (3.5%)	32 (3.4%)	18 (3.9%)
SPATIAL FACTORS			
Min. dist. (km) to drug shop			
Mean(SD)	1.15 (1.12)	1.16 (1.03)	1.13 (1.30)
Min. dist. (km) to gov't. health centre			
Mean (SD)	2.80 (1.72)	3.00 (1.76)	2.41 (1.57)
District			
Buliisa	499 (35.1%)	319 (33.4%)	180 (38.6%)
Pakwach	111 (7.8%)	0 (0%)	111 (23.8%)
Mayuge	810 (57.0%)	635 (66.6%)	175 (37.6%)

S3 Supplementary figures

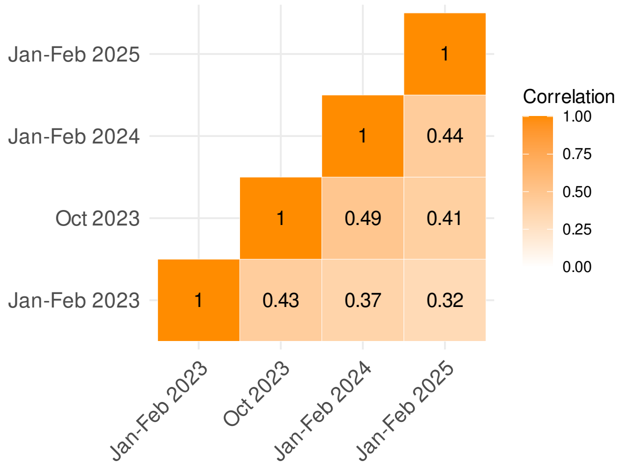

Fig. S1: **Spearman correlation heat map for attrition across timepoints.** Pairwise Spearman correlations of participant attrition status across study timepoints. Only statistically significant correlations ($p < 0.05$) are displayed.

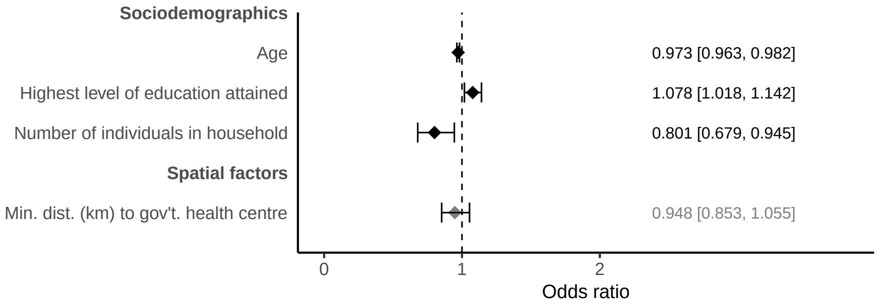

Fig. S2: **Model for true drop-outs.** Logistic regression model for true drop-outs who only attended the baseline timpoint ($n = 2828$) with 95% confidence intervals calculated using HH-level clustered standard errors (number of HH clusters = 1445). VIFs<10 for all variables. AUC for 10-fold cross-validation was 0.63. Results marked with a black diamond were significant, and those marked in grey were non-significant.

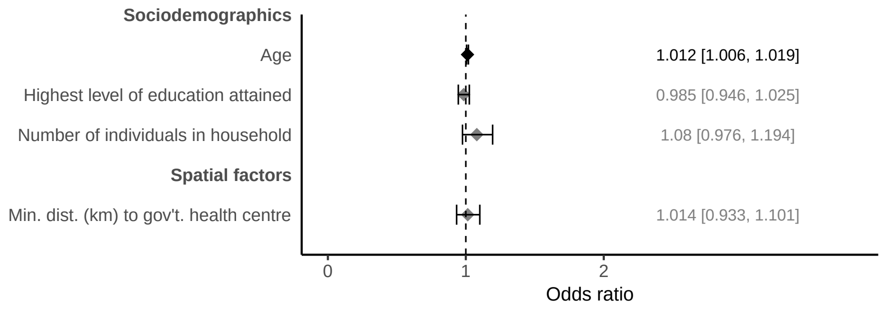

Fig. S3: **Overall rejoiner model.** Logistic regression model for rejoiners ($n = 1341$) with 95% confidence intervals calculated using HH-level clustered standard errors (number of HH clusters = 910). VIFs<10 for all variables. AUC for 5-fold cross-validation was 0.54. 5-fold cross-validation was used due to the smaller sample size. Results marked with a black diamond were significant, and those marked in grey were non-significant.

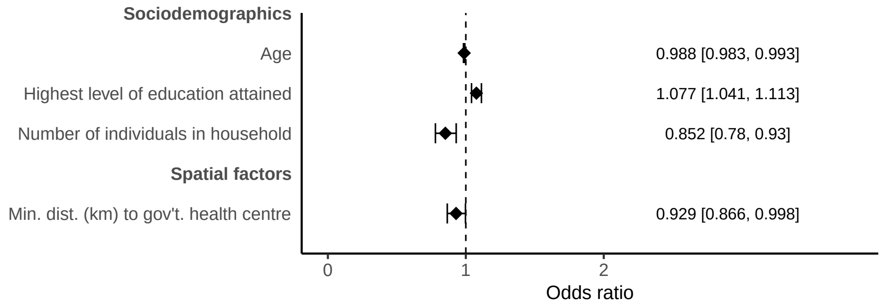

Fig. S4: **Model for attrition in Jan-Feb 2023.** Logistic regression model for attrition of study participants ($n = 2828$) in Jan-Feb 2023 with 95% confidence intervals calculated using HH-level clustered standard errors (number of HH clusters = 1445). VIFs<10 for all variables. AUC for 10-fold cross-validation was 0.58. Results marked with a black diamond were significant and those marked in grey were non-significant.

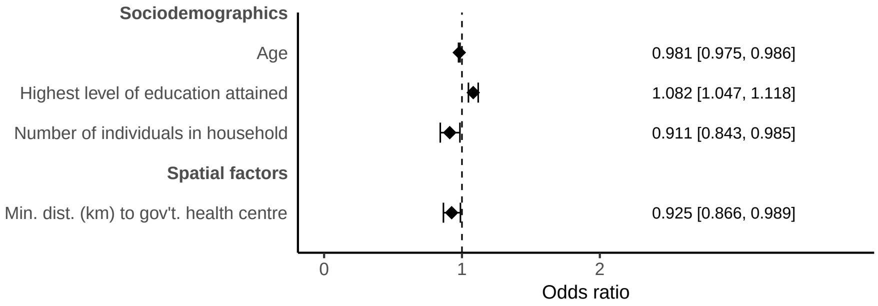

Fig. S5: **Model for attrition in Oct 2023.** Logistic regression model for attrition of study participants ($n = 2828$) in Oct 2023 with 95% confidence intervals calculated using HH-level clustered standard errors (number of HH clusters = 1445). VIFs<10 for all variables. AUC for 10-fold cross-validation was 0.6. Results marked with a black diamond were significant and those marked in grey were non-significant.

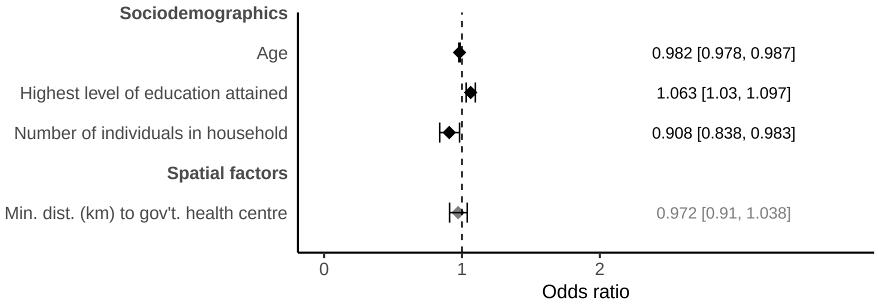

Fig. S6: **Model for attrition in Jan-Feb 2024.** Logistic regression model for attrition of study participants ($n = 2828$) in Jan-Feb 2024 with 95% confidence intervals calculated using HH-level clustered standard errors (number of HH clusters = 1445). VIFs<10 for all variables. AUC for 10-fold cross-validation was 0.58. Results marked with a black diamond were significant and those marked in grey were non-significant.

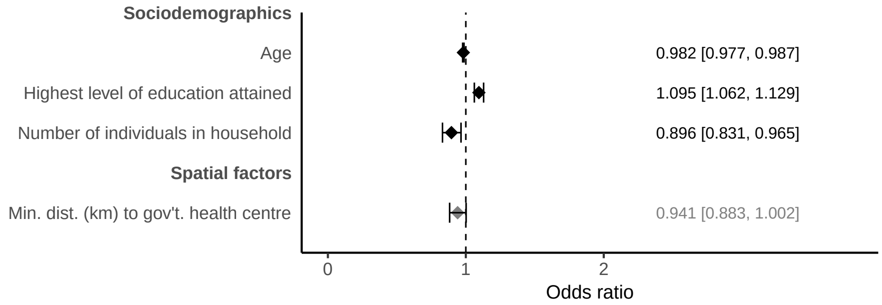

Fig. S7: **Model for attrition in Jan-Feb 2025.** Logistic regression model for attrition of study participants ($n = 2828$) in Jan-Feb 2025 with 95% confidence intervals calculated using HH-level clustered standard errors (number of HH clusters = 1445). VIFs<10 for all variables. AUC for 10-fold cross-validation was 0.6. Results marked with a black diamond were significant and those marked in grey were non-significant.

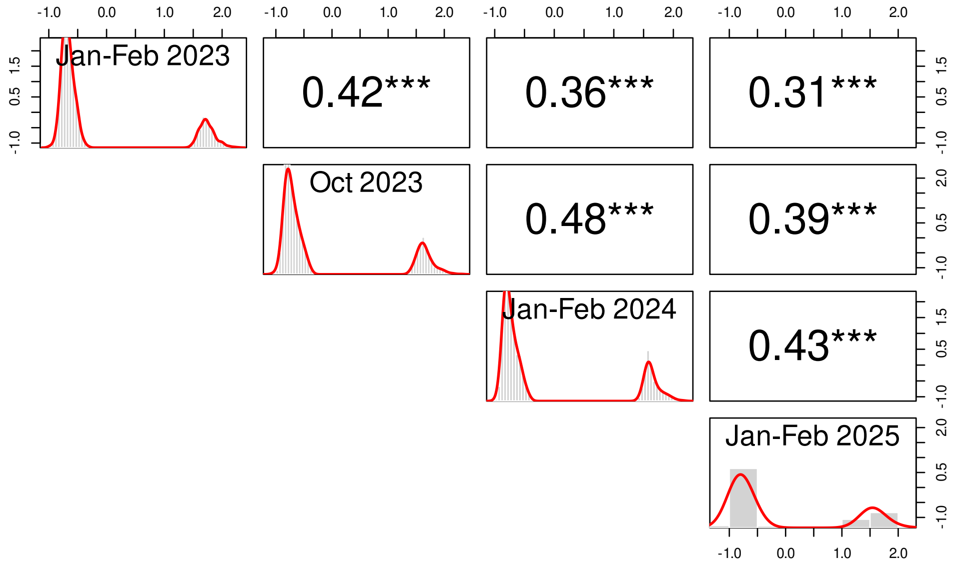

Fig. S8: **Correlation of residuals.** Pearson correlation coefficients between residuals of logistic regression models at the major study time points (Jan–Feb 2023, Oct 2023, Jan-Feb 2024 and Jan–Feb 2025). *** indicates p-value<0.001, demonstrating significant correlation at all time points.

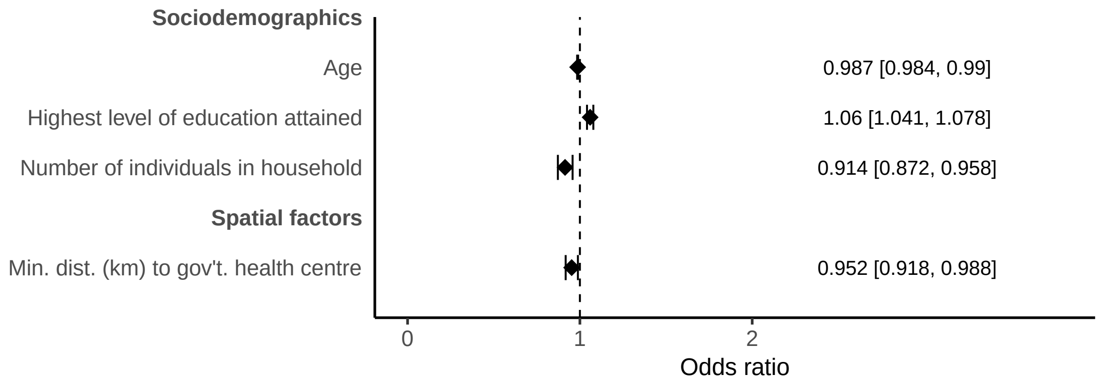

Fig. S9: **Negative binomial model for attrition count.** Negative binomial regression model predicting attrition count outcome for study participants ($n = 2828$), with 95% confidence intervals calculated using HH-level clustered standard errors (number of HH clusters = 1445). VIFs$<$10 for all variables. Model performance based on 10-fold cross-validation showed mean RMSE of 1.25. Results marked with a black diamond were significant and those marked in grey were non-significant.

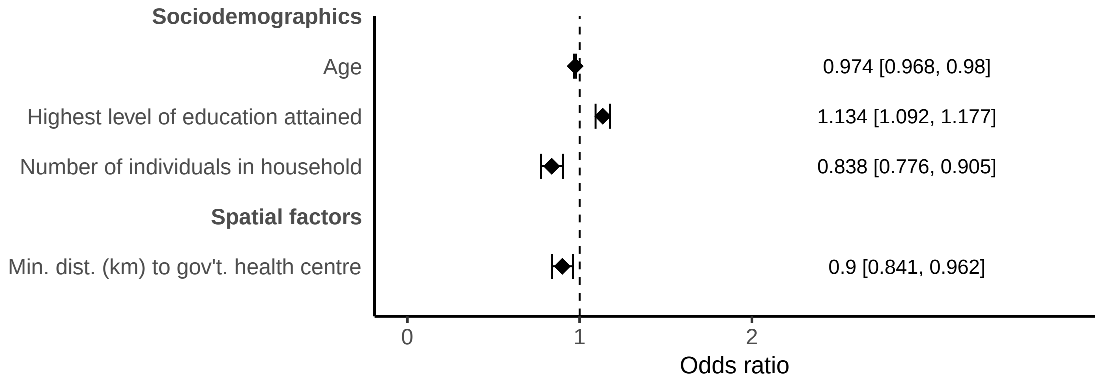

Fig. S10: **MLM with clusters at the individual and HH levels.** MLM model for recurrent attrition ($n = 11312$) with clusters at the individual-level (number of clusters = 2828) and HH-level (number of clusters = 1445). VIFs$<$10 for all variables. AUC for 10-fold cross-validation was 0.6. Results marked with a black diamond were significant and those marked in grey were non-significant. The adjusted individual-level ICC is 0.143, and the HH-level ICC is 0.425. The empty MLM ICCs for individual and HH levels are 0.163 and 0.422 respectively.

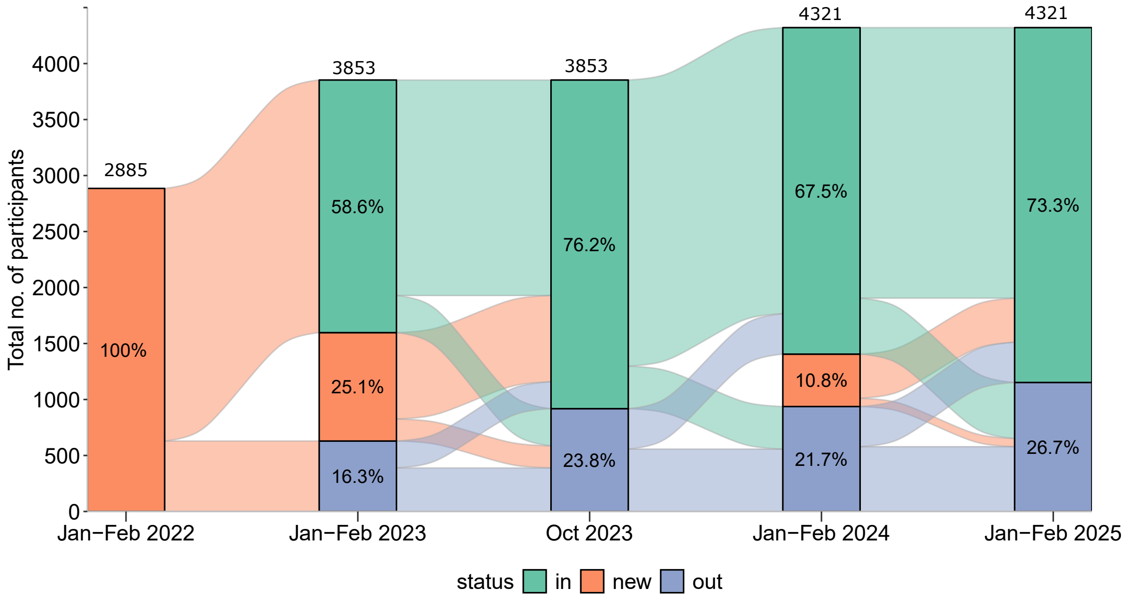

Fig. S11: **Study participation across timepoints.** Participant flows in and out of the study for all recruited participants, with major follow-up timepoints illustrated.

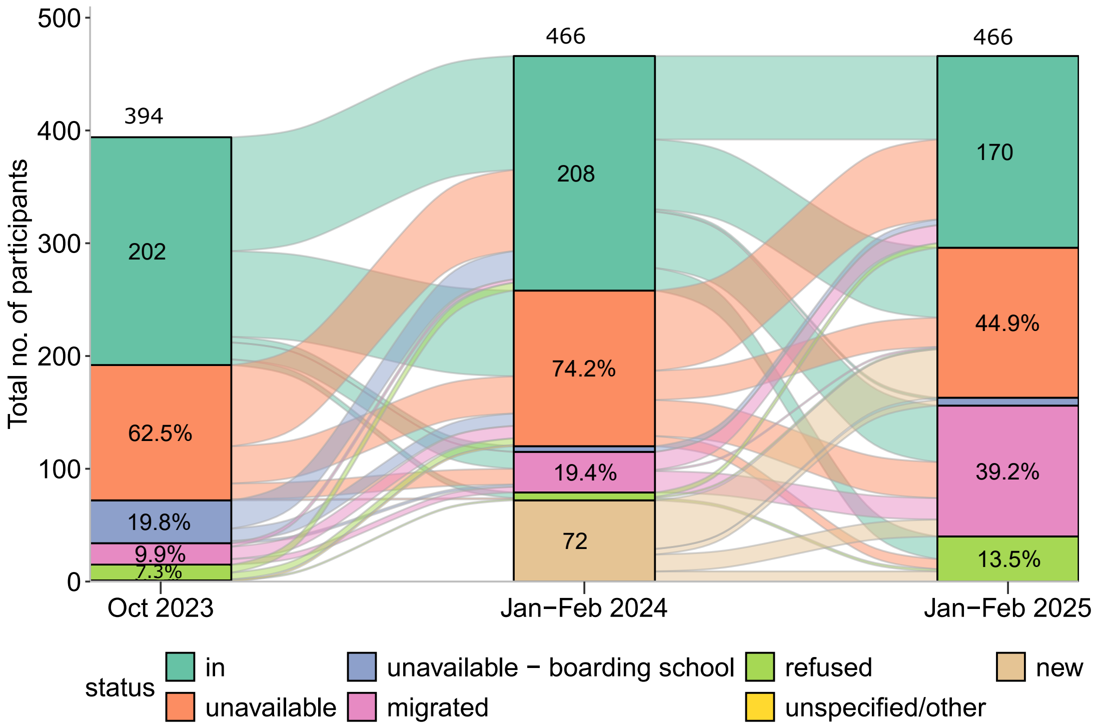

Fig. S12: **Attrition reasons for new recruits.** Attendance of new recruits who have had an attrition event across timepoints following expansions (n = 394 in Oct 2023, n = 466 in Jan-Feb 2024 and Jan-Feb 2025), along with reasons for attrition (n = 192 in Oct 2023, n = 186 in Jan-Feb 2024, n = 296 in Jan-Feb 2025). 0.5% (1/192) had 'other/unspecified' reason for attrition in Oct 2023, 2.7% (5/186) had reason 'unavailable-boarding school' for attrition in Jan-Feb 2024, 3.8% (7/186) refused to participate in Jan-Feb 2024, and 2.4% (7/296) had reason 'unavailable-boarding school' for attrition in Jan-Feb 2025.

References

1. Chami GF, Kontoleon AA, Bulte E, et al. Profiling nonrecipients of mass drug administration for schistosomiasis and hookworm infections: a comprehensive analysis of praziquantel and albendazole coverage in community-directed treatment in Uganda. Clinical Infectious Diseases. 2016;62(2):200-7.
2. World Health Organization. Improved sanitation facilities and drinking water sources;. [Accessed 6 Aug 2024]. Available: https://www.who.int/data/nutrition/nlis/info/improved-sanitation-facilities-and-drinking-water-sources.
3. World Health Organization, et al. Guidelines for drinking-water quality: incorporating the first and second addenda. World Health Organization; 2022.
4. Hijmans RJ, Van Etten J, Mattiuzzi M, et al.. Raster package in R;. [Accessed 20 Aug 2024]. Available: https://rspatial.org/raster/pkg/RasterPackage.pdf.
5. Richter J, Hatz C, Campagne G, et al.. Ultrasound in schistosomiasis: a practical guide to the standardized use of ultrasonography for the assessment of schistosomiasis-related morbidity: second international workshop held in Niamey, Niger, 22-26 October, 1996.;.
6. Anjorin S, Nabatte B, Mpooya S, et al. Epidemiology of periportal fibrosis and relevance of current Schistosoma mansoni infection within the context of repeated mass drug administration in rural Uganda: a population-based, cross-sectional study. The Lancet Microbe. 2024;5(12).
7. Katz N, Chaves A, Pellegrino J. A simple device for quantitative stool thick-smear technique in schistosomiasis mansoni. Revista do instituto de medicina tropical de São Paulo. 1972;14(6):397-400.
8. World Health Organization, et al. WHO guideline on control and elimination of human schistosomiasis. World Health Organization; 2022.
